# Proteomic profiling spotlights the molecular targets and the impact of the natural antivirulent umbelliferone on stress response, virulence factors, and the quorum sensing network of *Pseudomonas aeruginosa*

**DOI:** 10.3389/fcimb.2022.998540

**Published:** 2022-11-30

**Authors:** Thirupathi Kasthuri, Sivaraj Barath, Muruganandam Nandhakumar, Shunmugiah Karutha Pandian

**Affiliations:** Department of Biotechnology, Alagappa University, Karaikudi, India

**Keywords:** *Pseudomonas aeruginosa*, virulence factors, biofilm, anti-QS agents, umbelliferone, proteomic analysis, cytotoxicity analysis

## Abstract

*Pseudomonas aeruginosa* easily adapts to newer environments and acquires several genome flexibilities to overcome the effect of antibiotics during therapeutics, especially in cystic fibrosis patients. During adaptation to the host system, the bacteria employ various tactics including virulence factor production and biofilm formation to escape from the host immune system and resist antibiotics. Hence, identifying alternative strategies to combat recalcitrant pathogens is imperative for the successful elimination of drug-resistant microbes. In this context, this study portrays the anti-virulence efficacy of umbelliferone (UMB) against *P. aeruginosa*. UMB (7-hydroxy coumarin) is pervasively found among the plant family of *Umbelliferae* and *Asteraceae*. The UMB impeded biofilm formation in the *P. aeruginosa* reference strain and clinical isolates on polystyrene and glass surfaces at the concentration of 125 µg/ml. Global proteomic analysis of UMB-treated cells revealed the downregulation of major virulence-associated proteins such as RhlR, LasA, AlgL, FliD, Tpx, HtpG, KatA, FusA1, Tsf, PhzM, PhzB2, CarB, DctP, MtnA, and MscL. A functional interaction study, gene ontology, and KEGG pathway analysis revealed that UMB could modulate the global regulators, enzymes, co-factors, and transcription factors related to quorum sensing (QS), stress tolerance, siderophore production, motility, and microcolony formation. *In vitro* biochemical assays further affirmed the anti-virulence efficacy of UMB by reducing pyocyanin, protease, elastase, and catalase production in various strains of *P. aeruginosa*. Besides the antibiofilm activity, UMB-treated cells exhibited enhanced antibiotic susceptibility to various antibiotics including amikacin, kanamycin, tobramycin, ciprofloxacin, and cefotaxime. Furthermore, *in vitro* cytotoxicity analysis revealed the biocompatibility of UMB, and the IC_50_ value was determined to be 249.85 µg/ml on the HepG2 cell line. Altogether, the study substantiates the anti-virulence efficacy of UMB against *P. aeruginosa*, and the proteomic analysis reveals the differential expression of the regulators related to QS, stress response, and motility factors.

## Introduction

Bacterial adaptation to the host environment is one of the initial events of disease establishment in pathogenesis. Bacteria undergo a series of alterations in genetic events during their adaptation to the highly fluctuating environments of the host system. This highly dynamic and multifaceted process triggers bacteria to switch between two different life forms: a planktonic unicellular life phase and a sessile biofilm-encased multicellular life phase. The unicellular form favors free swimming of bacteria, and this physiological event is conducive to bacterial dispersion and colonization in a new environment, while the multicellular form favors the coordinated life forms and proliferation of bacteria within the biofilm matrix that protects the bacteria from hostile environments of the host system (Berlanga and Guerrero, 2016). The biofilm matrix-encased sessile cells are highly recalcitrant to antibiotics and become more tolerant. Among the biofilm-forming nosocomial infection pathogens, *Pseudomonas aeruginosa* is one of the deleterious opportunistic human pathogens.

*P. aeruginosa* is a ubiquitous, Gram-negative, monoflagellated pathogen that causes serious life-threatening infections, especially in those who have a weak immune system or individuals suffering from any disease. It is a common causative agent of bacteremia in burn victims, urinary tract infections in catheterized patients, ocular infections, and hospital-acquired pneumonia in ventilator-dependent patients (Bodey et al., 1983; Berra et al., 2010; Dave et al., 2020; Habboush et al., 2021; Kunwar et al., 2021; Newman et al., 2022). Furthermore, *P. aeruginosa* adapt to cystic fibrosis (CF) airways and survive as overpowering, pervasive, and ineradicable infectious agents until the patient dies (Döring et al., 2000). A major cause of morbidity and death in CF patients is recurrence of bacterial infections in the aberrant mucus layers (Khan et al., 1995; Malhotra et al., 2019; Henderson et al., 2022). To maintain appropriate mucus characteristics and homeostasis, the CF transmembrane conductance regulator (CFTR) protein controls the transfer of electrolytes and chlorides across epithelial cell membranes. In some genetic abnormalities, the CFTR protein’s lack of function accumulates unusually thick, dried, and sticky mucus layers on the airway epithelial cells of lungs (Folkesson et al., 2012; Stoltz et al., 2015; Kotnala et al., 2021). Subsequently, the patients become highly vulnerable to respiratory infections and *P. aeruginosa* is a noticeable dominant pathogen in the airways of CF patients (Grubb and Livraghi-Butrico, 2022). The complex regulatory genes of *P. aeruginosa* favor the bacterium to respond and adapt to various environments in addition to displaying a vast range of innate resistance to antibiotics (Stover et al., 2000).

In addition to the innate resistance mechanism of *P. aeruginosa*, the tendency of developing rapid resistance to various antimicrobial drugs worsens the treatment strategies (Pechère and Köhler, 1999; Montero et al., 2021), especially when the multicellular life form with thickened matrixome of bacteria develops antibiotic resistance (Karygianni et al., 2020). The *P. aeruginosa* extracellular secretome comprising four endopeptidases, elastase, alkaline proteinase, LasA protease, and lysine-specific endopeptidase, is implicated in the pathogenicity of the bacterium (Kessler et al., 1998). Furthermore, alginate polysaccharides and associated genes including *algL, algR* (*algU* and *algD*), *psl*, and *pel* are other major virulence traits of *P. aeruginosa* strains. These factors help in establishing the strongest biofilms and microcolonies on airways and lung surfaces (Lambert, 2002; Lee and Yoon, 2017). In addition to this, enzymes/proteins such as phospholipase C, exotoxins and cytotoxins, flagella and pili, pigments, and T3SS, T4SS, and T6SS modulate the production of virulence factors and biofilm formation in the host system (Azam and Khan, 2019; Qin et al., 2022).

Furthermore, the pathogenic traits of *P. aeruginosa* are synchronized by the hierarchical quorum sensing (QS) systems consisting of various regulators and signal molecules (Lee and Zhang, 2015). The canonical QS systems such as *las, rhl*, *pqs*, and *iqs* are well established in *P. aeruginosa* (Wang et al., 2016; Welsh and Blackwell, 2016; Veetilvalappil et al., 2022). Notably, proper folding and oligomerization of QS system-associated proteins majorly govern the expression and functional features of virulence factors (Heras et al., 2015). Hence, the bacterial machinery involved in the virulence factor production can be a potential drug target in hampering disease dissemination (García-Fernández et al., 2017; Kahler et al., 2018). Thus, studies contributing to elucidating the key molecular targets of bioactive molecules are of great importance in understanding the virulence-associated pathways and identification of potential drug candidates. Generally, anti-virulence medications interrupt the biosynthesis of virulence factors by impeding global regulators (Feng et al., 2014; Mühlen and Dersch, 2015). Moreover, Walz et al. (2010) demonstrated reduced bacterial virulence with increased pathogen clearance in anti-QS drug-treated animal models. Combining the anti-QS agents with conventional antibiotics enhances the antibiotic efficacy concomitant with lower healthcare costs, and this strategy is anticipated to be a primary treatment method in the near future. With this perspective, the present investigation aimed to substantiate the anti-QS efficacy of the plant-derived phytocompounds umbelliferone (UMB) against *P. aeruginosa.*

A benzopyrone group of coumarin derivative UMB is found in several plants and edible fruits (Ramalingam and Vaiyapuri, 2013). UMB is identified as a potential pharmaceutical agent with antihyperglycemic, antioxidant, antimicrobial, and antitumor activities (Mazimba, 2017). Swetha et al. (2019) reported the antibiofilm activity of UMB against *Staphylococcus epidermidis.* Though previous studies reported the various pharmacological effect of UMB, the present study is the first report on analyzing the anti-QS effect and delineating the molecular targets of UMB on WHO priority level I Gram-negative pathogen *P. aeruginosa.* The present investigation divulges the molecular mechanism of action of UMB on *P. aeruginosa* through global proteome analysis using liquid chromatography tandem mass spectrometry (LC-MS/MS) and further examines the cytotoxicity *via in vitro* study using HepG2 cell lines. In addition, the study substantiates the anti-virulence efficacy of UMB against various strains of *P. aeruginosa* isolated from clinical samples of human origin.

## Materials and methods

### Bacterial strains and culture conditions

*P. aeruginosa* PAO1 reference strain and clinical isolates (CI06, CI14, CI17, CI19, CI23, and CI24) used in this study were obtained from the microbial culture collection in the Department of Biotechnology, Alagappa University and cultured in Luria-Bertani (LB) agar and maintained at 4°C. A single colony of axenic culture from the stored plates was used to inoculate 2 ml of growth medium and incubated at 37°C overnight at 160 rpm and used as primary inoculum for all the assays. LB broth was used for biochemical assays and intracellular protein isolation from the control and treated groups. UMB (Sigma Aldrich, USA; MW: 162.14; CAS: 93-35-6; Lot # BCBQ1425V) was dissolved at 10 mg/ml concentration in methanol and stored at 4°C. The HepG2 cell line was procured from the National Centre for Cell Science, Pune and maintained in DMEM low-glucose medium.

### Determination of minimum biofilm inhibitory concentration of umbelliferone against *P. aeruginosa*

The minimum biofilm inhibitory concentration (MBIC) of UMB was determined using crystal violet (CV) staining assay in 24-well polystyrene plates (Adonizio et al., 2008). Initially, phytocompound was added to LB to final concentrations in the range of 15.6 µg/ml to 500 µg/ml in individual wells. Then, 1% bacterial inoculum (10^8^ CFU/ml) was added to each well. Wells containing growth medium alone and growth medium with inoculum were maintained as blank and inoculum control, respectively. The well plates were then incubated at 37°C for 24 h. After incubation (**Supplementary Figure 1A**), the absorbance was measured at OD_600_ using a spectrophotometer (Spectramax M3, USA). The planktonic cells were then removed, and the cells were gently washed three times with sterile distilled water to remove the loosely bound cells on the surface. Subsequently, 0.4% crystal violet was added to each well and the cells were allowed to absorb the stain at room temperature for 20 min. After staining, wells were washed with distilled water and then 30% glacial acetic acid (GAA) was added. The GAA elutes the stains absorbed by the biofilm matrix and, hence, the intensity of blue color of the GAA solution is reciprocal to the density of the biofilm matrix (**Supplementary Figure 1B**). The intensity of the blue color was read spectrophotometrically at OD_570_. Then, the percentage of growth inhibition was determined using the formula given below.

% of biofilm inhibition = (Control OD − Test OD/Control OD) × 100. [Test represents LB added with UMB and inoculum; Control represents LB with bacterial inoculum alone.]

### Assessing the effect of UMB on the growth of *P. aeruginosa* using resazurin-based metabolic viability assay

Metabolic viability assay was performed following the method described by Kasthuri et al. (2022). Initially, the experiment was carried out in 24-well plates, similar to the MBIC determination method. Resazurin was used as an indicator dye to assess the effect of UMB on the viability of *P. aeruginosa*. The metabolically active cells convert the oxidized form of resazurin to resorufin, which alters the resazurin’s innate blue color to pink. Briefly, after overnight incubation of 24-well assay plates, 160 µM (40 µl from 4 mM stock solution) resazurin indicator solution was added to each well and incubated for 1 h at 37°C. After incubation, the fluorescence intensity of the pink color (**Supplementary Figure 1C**) was measured at 560 nm and 590 nm excitation and emission range, respectively, using a spectrophotometer (Spectramax M3, USA).

### Growth curve analysis of *P. aeruginosa* control and umbelliferone treatment

Growth curve analysis was performed in order to understand the impact of UMB on the growth rate of *P. aeruginosa* following the method described by Valliammai et al. (2019). Initially, 50 ml of LB broth with and without 125 µg/ml of UMB was maintained as treated and untreated control groups, respectively. Methanol was added to the untreated control to understand the effect of vehicle on bacterial growth. Cefotaxime at the MIC of 156.25 µg/ml was used as a positive control. All three groups were added with the primary inoculum of 1% overnight culture of *P. aeruginosa* and incubated at 37°C with 160 rpm. Changes in absorbance at 600 nm were recorded to monitor the growth rate after every 2-h interval for 24 h, and the measurements were visualized as a line graph.

### Enumeration of planktonic and biofilm cells

Enumeration of planktonic and biofilm cells was carried out using the standard plate count method according to the protocol described by Adetunji and Odetokun (2012). The UMB at the concentration range of 15.6 µg/ml to 500 µg/ml was added to the glass tubes containing 2 ml of LB broth. Then, 1% of overnight culture (10^8^ CFU/ml) was added to each tube and incubated at 37°C for 24 h at 160 rpm. After incubation, planktonic cells were serially diluted and the 100 µl of 10^-9^ dilution was spread on LB agar in three technical replicates and incubated at 37°C for 24 h. For enumerating the biofilm cells, the planktonic culture was discarded and then gently washed with sterile saline (0.85%). The washing was carried out four times to remove the planktonic cells. The biofilm cells were then scraped off using 1-ml polystyrene tips with the aid of a micropipette and resuspended with 1000 µl of sterile saline. Then, the cells were serially diluted and the 100 µl of 10^-5^ dilution was spread on LB agar plates and incubated at 37°C for 24 h. After incubation, CFU was calculated for both planktonic and biofilm cells by counting the number of viable cells present in each dilution. Plates with too numerous to count colonies were considered to have 300 colonies when counting the viable cells. 

### Visualization of biofilm density on glass surfaces

The density of biofilm on the glass surface was visualized through ring biofilm assay and microscopic visualization. For the ring biofilm assay, the experiment was performed in glass test tubes as stated earlier in the CV staining assay. Initially, the tubes with 2 ml of LB broth containing the phytocompounds at the range of 15.6 µg/ml to 500 µg/ml were added with 1% overnight culture (10^8^ CFU/ml) and incubated at 37°C for 24 h at 160 rpm. After incubation, the culture (**Supplementary Figure 2A**) was removed and the tubes were gently rinsed with 3 ml of sterile distilled water by swirling and discarded. The process was repeated three times to remove the loosely bound cells and the biofilm matrix was stained with CV and the images were photographed. *P. aeruginosa* has a tendency to form a characteristic air–liquid interface biofilm and, hence, for microscopic visualization of *P. aeruginosa* biofilm on glass surfaces, the following protocol was carried out. Briefly, wells containing 1 ml of LB broth with 125 µg/ml of UMB and wells without treatment (added with 12.5 µl of solvent as a vehicle) were used as treated and control groups, respectively. Then, 1% overnight culture (10^8^ CFU/ml) was added to each well. The wells were then added with an approximately 1-cm^2^ glass piece diagonal to the broth surface (**Supplementary Figure 2B**) and incubated at 37°C for 24 h. After incubation, the slides were carefully taken with forceps and gently washed three times in sterile distilled water (**Supplementary Videos 1**–**3**) and then stained with 0.4% CV. The unabsorbed dyes were removed by washing the slides in sterile distilled water. After air drying at room temperature, the biofilm architecture of control and treated samples was micrographed using the light microscope (Nikon Eclipse 80i, USA).

### Evaluation of the effect of UMB on clinical isolates of *P. aeruginosa* biofilms

In general, clinical isolates from various infections differ in their virulence and phenotypic characteristics including biofilm formation and sensitivity to antibiotics. Hence, the biofilm inhibition potential and anti-QS effect of UMB on various clinical isolates were evaluated using CV staining and metabolic viability assays in 24-well MTP as described earlier. Clinical strains such as CI06, CI14, CI17, CI19, CI23, and CI24 were isolated from sputum, urine, and wound samples of patients and previously characterized by Sethupathy et al. (2016). The assays were performed with the primary inoculum of 1% overnight culture containing 1×10^8^ CFU/ml. After 24-h incubation (**Supplementary Figure 2C**), the biofilm density on the glass surface of the treated and untreated groups of six different clinical isolates was visualized in the light microscope by following the above-described method.

### Analysis of the impact of UMB on the proteome of *P. aeruginosa*

#### Protein extraction and purification

Initially, 125 µg/ml of the UMB-treated and untreated group (added with vehicle control) of *P. aeruginosa* were grown in 100 ml of LB broth for 18 h with the OD_600_ of 0.8–1.0. The planktonic cells were then harvested by centrifugation at 8,000 rpm for 10 min at 4°C. The cell pellets were washed twice with 20 mM Tris (pH 8.0), and then, the intracellular proteins of the treated and untreated groups of *P. aeruginosa* were isolated using sonication by following the method described by Shrestha et al. (2012). Initially, the washed cells were resuspended in 20 mM Tris (pH 8.0) with 1 mM phenylmethanesulfonyl fluoride (PMSF) and kept in an ice box to prevent the heating of the sample during sonication. The cells were then sonicated with Vibra Cell VCX 750 probe sonicator containing a 422-probe with a tip diameter of 3 mm (Sonics and Materials, Newtown, CT, USA). An ultrasonic frequency of 20 kHz was applied with the parameters including 35% amplitude, 35°C probe tip temperature, and 10 s on and off pulse cycle for 40 min. After sonication, the cell debris was removed by centrifugation at 8,000 rpm for 5 min and the supernatant was quantified using Bradford assay. Then, the protein concentration of the treated and untreated groups was normalized to 1 mg/ml using 20 mM Tris (pH 8.0) and further processed for LC-MS/MS analysis.

#### Nano-LC-MS/MS analysis

Relative protein quantification of control and treated samples was performed using ESI-nano LC-MS/MS analysis [a nano ACQUITY UPLC^®^ chromatographic system (Waters) coupled with a Quadrupole Time-of-Flight (Q-TOF) mass spectrometer (SYNAPT-G2, Waters)] as described by Aneesh Kumar et al. (2021). In short, the samples were reduced using 100 mM 1,4-dithiothreitol (Sigma Aldrich) and then alkylated using 200 mM iodoacetamide (Sigma Aldrich) and digested overnight with MS grade trypsin (Sigma Aldrich) in a 1:25 ratio (1 µg of trypsin to 25 µg of protein), and the samples were injected in duplicate. The obtained mass spectra were then analyzed using Progenesis QI for Proteomics V4.2 (Non-Linear Dynamics, Waters) with a maximum of one missed cleavage per peptide. UniProt reviewed entry database source was used for identification of *P. aeruginosa* proteins.

#### *In silico* analysis and functional insights into the proteome of UMB-treated *P. aeruginosa*

Protein–protein interaction analysis of differentially regulated proteins was performed using the online database STRING, version 11.5, with a medium confidence interaction score (0.400). The interacting partners were grouped into various functional clusters using the K-means clustering algorithm. Initially, interaction analysis for the differentially regulated proteins with statistical significance (fold change > ± 1 and *p* ≤ 0.05) was performed, and the network was constructed using Cytoscape v3.9.1. Functional enrichment analysis was performed using DAVID bioinformatic database-based gene enrichment analysis (GEA), and the proteins belonging to the various functional clusters and GO terms were compared and visualized as chord diagram and bar graph, respectively, using R version 4.1.2. KEGG reference pathways and *P. aeruginosa* specific metabolic pathways were used to identify the functional insights into differentially regulated proteins on metabolic pathways of *P. aeruginosa.*

### Assessment of the impact of UMB on virulence traits of *P. aeruginosa*

*In vitro* biochemical assays help in assessing the impact of UMB on virulence-mediated phenotypic traits of *P. aeruginosa.* Although LC-MS/MS analysis provides insights into the differential expression of various genes/proteins at the molecular level with precise quantification, biochemical assays further substantiate the observable variation in phenotypic traits of the control and treated groups. All the assays were carried out for both reference strain and clinical isolates from the 18-h-grown culture of control (added with vehicle control) and treated (MBIC concentration of UMB) groups using the methodologies described by Sethupathy et al. (2016) unless otherwise mentioned. Initially, the overnight grown culture was adjusted to an OD_600_ of 0.2 using LB broth and used as primary inoculum. The control and treated group in LB with 1% of primary inoculum was incubated at 37°C for 18 h at 160 rpm. After incubation, the supernatant and cell pellets were collected by centrifugation at 10,000 rpm for 10 min. For enzymatic assays, the protein concentration of intracellular lysates was initially normalized with 20 mM Tris (pH 7.4) after the Bradford quantification assay.

#### Pyoverdine assay

Initially, cell-free culture supernatant (CFCS) was collected from the 18-h-grown culture of the UMB-treated and untreated groups of *P. aeruginosa.* The CFCS was then diluted with 0.1 M Tris-HCl (pH 8.0). Then, the amount of pyoverdine produced in the control and treated groups was determined by measuring the absorbance at 405 nm.

#### Pyocyanin assay

Quantification of pyocyanin from the CFCS of control and treated cells was performed by following the method described by Das et al. (2015). Initially, the CFCS of the control and treated groups was collected by centrifugation at 10,000 rpm for 10 min. The CFCS was then filtered through a 0.2-µm membrane filter, and the concentration of pyocyanin was determined by measuring the absorbance at 691 nm, which is the *λ*_max_ of pyocyanin.

#### Casein degrading protease assay

An equal volume of CFCS from *P. aeruginosa* control and UMB-treated culture was incubated with substrate buffer (3 mg/ml azocaesin in 0.05 mM Tris hydrochloride, pH 7.5, and 0.5 mM CaCl_2_) for 15 min at 37°C. After incubation, an equal volume of 10% trichloroacetic acid (TCA) was added to stop the proteolytic activity, and then, the samples were centrifuged for 10 min at 10,000 rpm, and the absorbance was measured at 440 nm.

#### LasB elastase assay

The CFCS from *P. aeruginosa* control and treated culture was mixed with elastase substrate buffer (20 mg/ml elastin Congo red in 100 mM Tris hydrochloride and 1 mM CaCl_2_, pH 7.5) in a 1:9 ratio and incubated at 37°C for 3 h with agitation. The remaining unused elastin Congo red in the reaction mixture was pelleted down by centrifugation at 10,000 rpm for 10 min and then the absorbance of soluble Congo red in the supernatant was measured at 495 nm.

#### Catalase assay

*P. aeruginosa* was pelleted by centrifugation at 12,000 rpm for 10 min at 4°C after being cultured in the absence and presence of UMB for 18 h at 37°C. The pellets were then washed and resuspended in ice-cold 50 mM potassium phosphate (KPi) solution (pH 7.0). Samples were sonicated on ice as mentioned earlier to extract crude cellular protein. After sonication, cell debris was removed by centrifugation at 13,000 rpm for 20 min and the protein concentration was normalized with PBS (pH 7.0) after quantification with Bradford assay. Catalase activity was determined by incubating 1 ml of substrate buffer (18 mM H_2_O_2_ in 50 mM potassium phosphate buffer, pH 7.0) with 1 mg of protein from the control and treated groups at 27°C. H_2_O_2_ decomposition was observed for 1 min at 240 nm, and specific activity was estimated using the formula: Specific activity = (OD_240_/min × 3,000)/(43.6 (Mϵ) × mg protein).

#### SOD activity assay

The protein samples isolated from 18-h-grown cultures of the treated and untreated groups of *P. aeruginosa* were normalized with Bradford assay. Then, 1 mg of protein from all the test and control groups was incubated with 5 μl of 10 mM pyrogallol in 10 mM HCl with 1 ml of oxygenated reaction buffer (50 mM Tris-HCl and 1 mM EDTA, pH 8.0). Oxidation of pyrogallol was monitored for 1 min at room temperature at 320 nm and specific activity was calculated as U/mg of protein using the formula: Specific activity = (OD_240_/min × 3,000)/(43.6 (Mϵ) ×mg protein)

#### ROS accumulation assay

*P. aeruginosa* was cultured for 18 h at 37°C with 160 rpm in the presence and absence of UMB. After incubation, the absorbance of the control and treated samples was adjusted to 0.4 at 600 nm using LB broth. Then, 100-μl aliquots were centrifuged for 5 min at 5,000 rpm, washed in phosphate-buffered saline (PBS), and resuspended in 1 ml of PBS. Each well of a 96-well plate containing 100 μl of 50 μM 2’,7’-dichlorofluorescein diacetate (H_2_DCF-DA) was mixed with 100 μl of control and treated cells and incubated at 37°C for 30 min. ROS-associated fluorescence was measured by the fluorescence plate reader (SpectraMax 3, Molecular Devices, Sunnyvale, USA) at excitation 485 nm and emission 520 nm.

#### H_2_O_2_ sensitivity assay

H_2_O_2_ sensitivity assay was performed to understand the impact of UMB on the catalase production of test strains following the method described by Zhang et al. (2012). Initially, 3-h-grown *P. aeruginosa* culture containing 10^8^ CFU/ml was spread on LB agar plates supplemented with MBIC of UMB. LB agar without treatment was used as control. Then, a sterile filter disc (10 mm in diameter) was placed at the center of the plates and loaded with 25 µl of 30% H_2_O_2_ and incubated at 37°C for 24 h. After incubation, the zone of clearance was measured to analyze the H_2_O_2_ sensitivity pattern of the *P. aeruginosa* strains.

### Antibiotic susceptibility test

An antibiotic susceptibility test was performed following the method described by Swetha et al. (2019) with slight modification for reference strains and clinical isolates. A 3-h-grown culture of test strain was spread on Muller Hinton agar plates supplemented with or without MBIC of UMB using the sterile swab. Then, the antibiotic discs such as kanamycin 30 (K30), cefotaxime 30 (C30), amikacin 30 (AK30), ciprofloxacin 5 (CIP5), and tobramycin 10 (TOB10) (HiMedia Laboratories, India) were placed on the agar plates using sterile forceps. The plates were then incubated at 37°C for 24 h and the zone of inhibition was measured using the HiAntibiotic zone scale (Hi-Media Laboratories, India) and the plates were documented using GelDoc XR+ (Bio-Rad).

### Evaluating the differential expression of virulence factors through quantitative real-time PCR

Gene expression analysis of the treated and untreated groups was performed following the method described by Swetha et al. (2019). Initially, primers for *P. aeruginosa* virulence-associated genes *algL, clpB, clpP2, dctP, fliD, fusA1, katA, lasA, mscL, phzM, rhlR, sodB*, and *tpx* and the housekeeping gene *rpsL* were designed using primer3 software. The details of oligomers used in this study are listed in **Supplementary Table 1**. The designed primer sequences were procured from the manufacturer, Sigma-Aldrich Chemicals Private Limited, India. Then, the mRNA was isolated from 18-h-grown culture of the control and UMB-treated groups of *P. aeruginosa* following the Trizol method using the TRI reagent (Sigma Aldrich, USA). The isolated mRNA was quantified using a spectrophotometer (DenoVix DS-11 FX+, USA) and the concentration was normalized with RNase-free water. The transcripts were then converted to cDNA using a QunatiTect reverse transcription kit (Qiagen, USA). Then, the reactions were performed with SYBR Green master mix (Qiagen, USA) in 0.1-ml Fast Optical 96-well reaction plates (MicroAmp, Applied Biosystems, USA) following the protocol described by the manufacturer. The amplification of nucleic acids in the reaction mixture was performed in a thermal cycler (7500 Real-Time PCR system, OPTIPLEX 755, Applied Biosystems) with the set temperature profiling of initial denaturation at 95°C for 5 min followed by 30 cycles of denaturation at 95°C for 1 min, annealing at 55°C for 1 min, extension at 72°C for 1 min, and final extension at 72°C for 5 min. The amplification was monitored with the real-time camera in the thermal cycler. The cycle threshold values (Ct) obtained from the 7500 software V2.0.3 were normalized with the Ct value of *rpsL* (ΔCt), and the differential gene expression was analyzed using the comparative threshold method (ΔΔCt). Relative fold change in log2 was calculated to determine the differential expression of virulence genes in the treated group.

### Evaluation of cytotoxicity profile of UMB using HepG2 cell lines

**Figure 1:**
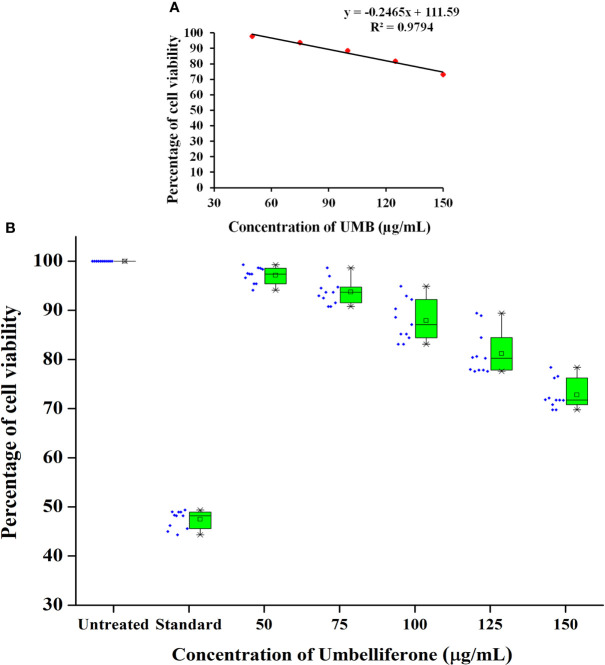
Cytotoxicity profile of various concentrations of UMB on HepG2 cells. MTT-based metabolic viability assay profile in understanding the effect of UMB on HepG2 cells after 24-h treatment **(A)**. Based on the assay, IC_50_ value is determined to be 249.85 µg/ml. Percentage of cell viability at various concentration of UMB on HepG2 **(B)** and the results reveal the non-adverse effect of UMB on HepG2 cells up to 150 µg/ml. Error bar represents the standard deviation from four independent experiments (*n* = 11).

The MTT assay was carried out to evaluate the cytotoxicity profile of UMB on HepG2 cell lines (NCCS, Pune, India) as described in Mosmann (1983). Initially, a 96-well plate containing DMEM low-glucose medium was seeded with 200 μl of HepG2 (NCCS, Pune, India) cell suspension (approximately 20,000 cells per well). The cells were allowed to grow for 24 h. Cells were then treated with UMB at the concentration range of 50, 75, 100, 125, and 150 µg/ml. Doxorubicin at 5 µM/ml was used as a positive control. Wells with medium alone and the medium seeded with cells were used as blank and control, respectively. The plate was then incubated 24 h at 37°C in a 5% CO_2_ atmosphere (CO_2_ incubator, Healforce, China). After incubation, spent medium was removed and the wells were washed twice with phosphate buffered saline (pH 7.2). Then, wells were added with MTT [3-(4,5-dimethylthiazol-2-yl)-2,5-diphenyl tetrazolium bromide; # 4060 HiMedia] reagent to a final concentration of 0.5 mg/ml of total assay volume. Plates were then wrapped with aluminum foil and then incubated for 3 h at room temperature. The MTT reagent was then removed and the wells were added with 100 μl of DMSO. The MTT formazan crystals were dissolved by gentle stirring and then the absorbance of eluted formazan was read at 570 nm and the percentage of cell viability was calculated from the mean absorbance using the following formula: % cell viability = (Mean absorbance of treated cells/Mean absorbance of untreated cells) × 100. The IC_50_ value was determined using linear regression equation i.e., *y* = *mx* + *c*. Here, *y* = 50, and *m* and *c* values were derived from the graph as given in **Figure 1A**. Direct microscopic observation of control and treated HepG2 cells after 24-h incubation was micrographed with the inverted binocular biological microscope (CKX415F, Olympus, Japan).

### Statistical analysis

Biofilm quantification assays, validation methods, and biochemical assays were performed in experimental and biological triplicates. Samples for mass spectrometric quantification were prepared in experimental and biological triplicates, and the LC-MS/MS analysis was performed in two technical replicates of pooled samples. The statistical analysis of mass spectrometric data was performed using Progenesis QI for Proteomics V4.2 (Non-Linear Dynamics, Waters). The values were expressed as mean ± standard deviation. Gene expression analysis with Real-time PCR was performed in two experimental replicates and biological triplicates, and the statistical analysis was performed using paired, two-tailed, *t*-test. MTT assay was performed in experimental and biological triplicates. Statistical analyses for biochemical assays were performed using SPSS Statistics V23.0 (SPSS Ltd, Hong Kong) software package. A Dunnett’s-ANOVA test was used to compare the treated and control groups. *p*-value < 0.05 was considered statistically significant for all the *in vitro* experiments.

## Results

### Effect of UMB on *P. aeruginosa* biofilm formation

**Figure 2:**
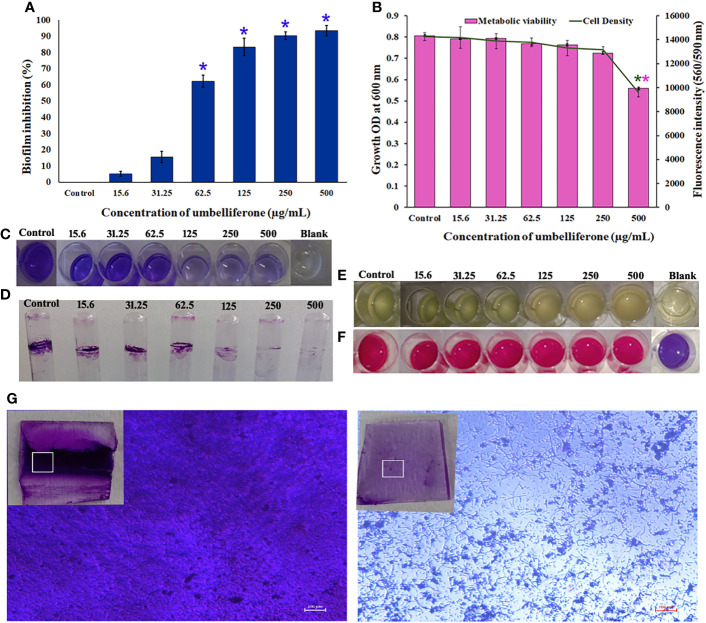
Effect of UMB on biofilm formation and growth of *P. aeruginosa* PAO1. UMB at 125 μg/ml showed 80% biofilm inhibition without significant alteration on the cell’s viability **(A)**. Observed growth OD and fluorescent intensity represent the amount of cell density and metabolic viability of *P. aeruginosa* at each tested concentration of UMB **(B)**. * & ** Represent significant reduction in biofilm formation (p < 0.05) & growth intensity (p < 0.01) respectively (n = 9). Representative images of 24-well plate CV staining assay depicting reduced biofilm formation upon treatment with UMB in a concentration-dependent manner **(C)**. Representative images of well plate showing bacterial density **(E)** and metabolic viability **(F)** at various concentrations of UMB. Ring biofilm assay demonstrating concentration-dependent decrease in biofilm formation of the treated cells **(D)**. Micrograph of biofilm cells of treated and untreated cultures (scale bar: 100 μm, magnification: 200×). Insets represent the crystal violet-stained biofilm cells on glass surface at the air–liquid interface of the control and treated groups **(G)**.

**Figure 3:**
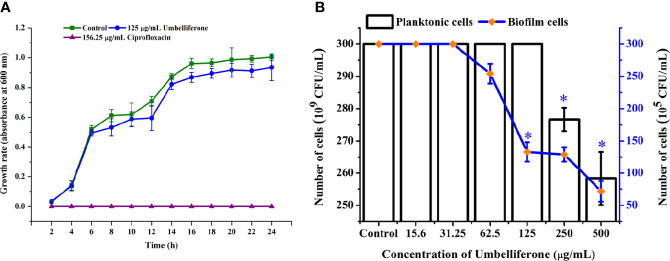
Effect of UMB (at MBIC) on the growth of *P. aeruginosa* PAO1. Growth curve analysis of*P. aeruginosa* control, with 125 μg/ml of UMB and 156.25 μg/ml of cefotaxime treatment showing no adverse effect of UMB (at 125 μg/ml) on the growth of *P. aeruginosa* at various time scales **(A)**. Enumerating the planktonic and biofilm cells of UMB-treated groups demonstrating non-adverse effect of UMB on planktonicgrowth of P. aeruginosa at test concentrations. Similarly, the biofilm cells are significantly decreased at MBIC of UMB **(B)**. Error bar represents the standard deviation (n = 9). * Denotes statistical significance with p-value < 0.05.

**Figure 4:**
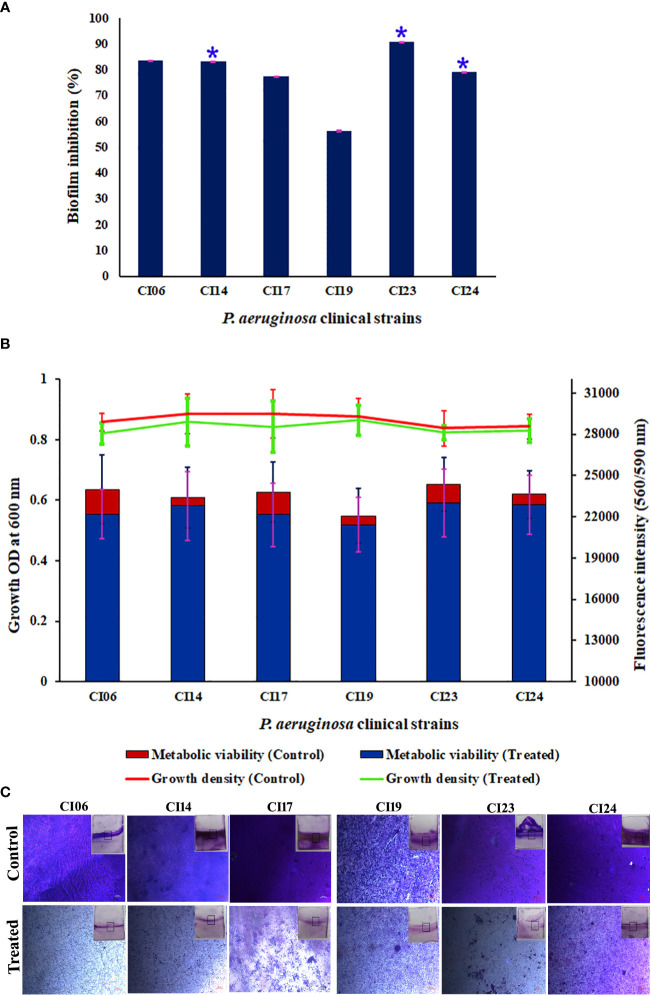
Effect of UMB (at 125 µg/ml) on biofilm formation and growth of various *P. aeruginosa* clinical isolates. Percentage of biofilm inhibition of CI06, CI14, CI17, CI19, CI23, and CI24 in CV staining assay showing the decreased biofilm formation in treated cells **(A)**. * represents significant reduction in biofilm formation with *p* < 0.05 (*n* = 9). Growth OD and fluorescent intensity represent the amount of cell density and metabolic viability of *P. aeruginosa* clinical isolates at 125 µg/ml of UMB **(B)**. Micrograph of biofilm cells of control and UMB treated cultures of *P. aeruginosa* clinical isolates (scale bar: 20 μm, magnification: 200×) **(C)**. Error bar represents the standard deviation from three independent analysis (*n* = 9).

The antibiofilm activity of UMB against *P. aeruginosa* was assessed using CV-based biofilm biomass quantification assay. Obtained results exemplified that the UMB treatment reduced the *P. aeruginosa* biofilm density in a concentration-dependent manner (**Figures 2A, C**). The MBIC of UMB was determined as 125 µg/ml, at which a maximum of 80% biofilm inhibition was observed. Significant reduction on *P.* aeruginosa viability was not observed in UMB-treated cells up to MBIC (**Figures 2B, E**). Viability in UMB-treated cells was evaluated using alamarBlue^®^-based metabolic viability assay (**Figure 2B**). In the alamarBlue^®^ assay, resazurin is reduced to resorufin by the electron transport system of viable cells, which turns the original blue of resazurin to pink (**Figure 2F**). The intensity of pink is reciprocal to the total number of viable cells in the samples (Selvaraj et al., 2019). The observed results confirmed the non-bactericidal antibiofilm activity of UMB against *P. aeruginosa*. In addition, the ring biofilm assay result demonstrated a concentration-dependent biofilm reduction in UMB-treated cells (**Figure 2D**). Similarly, light microscopic analysis also exhibited the reduced level of biofilm formation on glass slides of UMB treatment compared to the untreated control cells, which confirms the antibiofilm activity of UMB against *P. aeruginosa* (**Figure 2G**). Furthermore, no significant alteration in growth rate was observed in both UMB-treated and control cultures of *P. aeruginosa* (**Figure 3A**). Furthermore, the number of viable cells of planktonic and biofilm counterparts was enumerated using standard plate count method and the results of planktonic cell count demonstrated the presence of viable cells in all the test concentration of UMB without any significant alteration in the growth rate. The number of cells was observed to be decreased in biofilm cells upon increasing concentration of UMB treatment (**Figure 3B**). In addition, the impact of MBIC of UMB on biofilm formation of *P. aeruginosa* clinical isolates was evaluated and the results are provided in **Figures 4A–C**. The biofilm inhibition assay results showed 83.5 ± 0.05%, 83 ± 0.03%, 77.5 ± 0.08%, 56.3 ± 0.3%, 90.98 ± 0.04%, and 79 ± 0.02% reduction in biofilm formation of CI06, CI14, CI17, CI23, and CI24, respectively (**Figure 4A**) without any significant alteration in growth and metabolic activity of clinical isolates (**Figure 4B**). Similarly, the light microscopic analysis also revealed the reduction of biofilm density in treated groups of clinical isolates on the glass surfaces (**Figure 4C**). Collectively, the observed results substantiate the biofilm inhibition potential of UMB on *P. aeruginosa* strains.

### UMB differentially regulates the expression of intracellular proteome of *P. aeruginosa*

Differential expression in the proteome of the UMB-treated and untreated groups of *P. aeruginosa* was assessed through nano LC-MS/MS analysis. A total of 619 proteins were identified at various mass values ranging from 6,534.5 Da to 245,815.5 Da. Among the identified proteins, 173 and 358 were upregulated and downregulated respectively, and 85 remained unchanged (**Supplementary File 1**). From these differentially expressed proteins, 16 upregulated proteins and 34 downregulated proteins were picked based on the fold change ≥ 1 with *p* ≤ 0.05 as listed in **Tables 1**, **2**.

**Table 1:** List of proteins identified as upregulated from the expression profile of 619 proteins of UMB-treated and untreated *P. aeruginosa*.

UniProt accession no.	Description	ANOVA (*p*)	Max fold change
**Q9I0A2**	50S ribosomal protein L20 RplT	0.046	*1.757*
**Q9I1M0**	Lipoamide acyltransferase component of branched-chain alpha-keto acid dehydrogenase complex BkdB	0.040	*1.820*
**Q9I502**	Proline–tRNA ligase ProS	0.047	*1.125*
**Q9I2U1**	ATP-dependent Clp protease proteolytic subunit 1 clpP1	0.004	*3.201*
**P40947**	Single-stranded DNA-binding protein Ssb	0.003	*2.022*
**Q9HWE7**	50S ribosomal protein L5 RplE	0.007	*1.262*
**Q9HWE0**	50S ribosomal protein L22 RplV	0.010	*1.733*
**Q9HWD1**	30S ribosomal protein S7 RpsG	0.017	*1.692*
**P22608**	Type IV pilus assembly ATPase PilB	0.020	*3.200*
**Q9HVL6**	50S ribosomal protein L21 RplU	0.020	*1.928*
**Q9HZZ2**	Elongation factor P Efp	0.021	*2.674*
**Q9HWE3**	50S ribosomal protein L29 RpmC	0.033	*26.692*
**Q9HV46**	Transcription elongation factor GreA	0.034	*1.478*
**Q9HWE9**	30S ribosomal protein S8 RpsH	0.040	*3.023*
**Q9HT16**	ATP synthase subunit b AtpF	0.050	*1.469*

**Table 2:** List of proteins identified as downregulated (*p* ≤ 0.05 and *F* ≥ 1) from the expression profile of 619 proteins of UMB-treated and untreated *P. aeruginosa*.

UniProt accession no.	Description	ANOVA (*p*)	Fold change
**Q889C2**	Chaperone protein ClpB	0.02	1.59
**B3PLM6**	Chaperonin GroEL	0.01	1.89
**Q88FB9**	Chaperone protein HtpG	0.01	2.78
**Q4ZNN4**	Catalase KatA	0.01	1.65
**P57668**	Thiol peroxidase Tpx	0.03	1.24
**Q3KHB1**	Elongation factor Tsf	0.01	1.61
**Q4ZMP1**	Elongation factor G FusA1	0.05	3.54
**B1J479**	Alginate lyase AlgL	0.01	1.90
**Q02L47**	Phenazine biosynthesis protein PhzB2	0.05	1.37
**Q9HWH2**	Phenazine-1-carboxylate N-methyltransferase PhzM	0.02	2.18
**P54292**	Regulatory protein RhlR	0.01	1.66
**Q02L18**	Protease LasA	0.02	2.26
**Q87WP4**	Carbamoyl-phosphate synthase large chain CarB	0.03	1.71
**Q9I0K9**	Adenylosuccinate lyase PurB	0.03	2.49
**Q59637**	Pyruvate dehydrogenase E1 component AceE	0.02	1.67
**B7VB85**	Glutamine–tRNA ligase GlnS	0.03	2.00
**Q9HVM4**	Isoleucine–tRNA ligase IleS	0.00	2.63
**Q4ZQ92**	Methylthioribose-1-phosphate isomerase MtnA	0.02	1.18
**Q9HU18**	C4-dicarboxylate-binding periplasmic protein DctP	0.00	1.57
**A6VC02**	Large-conductance mechanosensitive channel MscL	0.03	2.72
**Q9K3C5**	B-type flagellar hook-associated protein 2 FliD	0.03	1.63
**Q88FB2**	Succinate–CoA ligase SucC	0.00	1.51
**Q8XVS2**	Arabinose import ATP-binding protein AraG	0.01	1.96
**B7UVT6**	Probable cytosol aminopeptidase PepA	0.02	2.71
**B7V8B5**	UPF0250 protein PLES_09781	0.02	2.31
**Q8XWT3**	Pantothenate synthetase PanC	0.02	1.79
**Q1I2H5**	tRNA modification GTPase MnmE	0.02	4.14
**P47203**	Cell division protein FtsA	0.02	1.24
**Q88FY7**	Porin-like protein NicP	0.02	6.69
**Q9HTB6**	GDP-6-deoxy-D-mannose reductase Rmd	0.02	1.49
**A6VCE7**	Ketol-acid reductoisomerase (NADP (+))	0.04	1.42
**Q9HT95**	Acetyltransferase PA5475	0.05	1.70
**C3K6G5**	Probable cytosol aminopeptidase PepA	0.05	1.45

### PPI network analysis reveals that the differentially regulated proteins interact with the proteins involved in virulence, quorum sensing, and stress response

**Figure 5:**
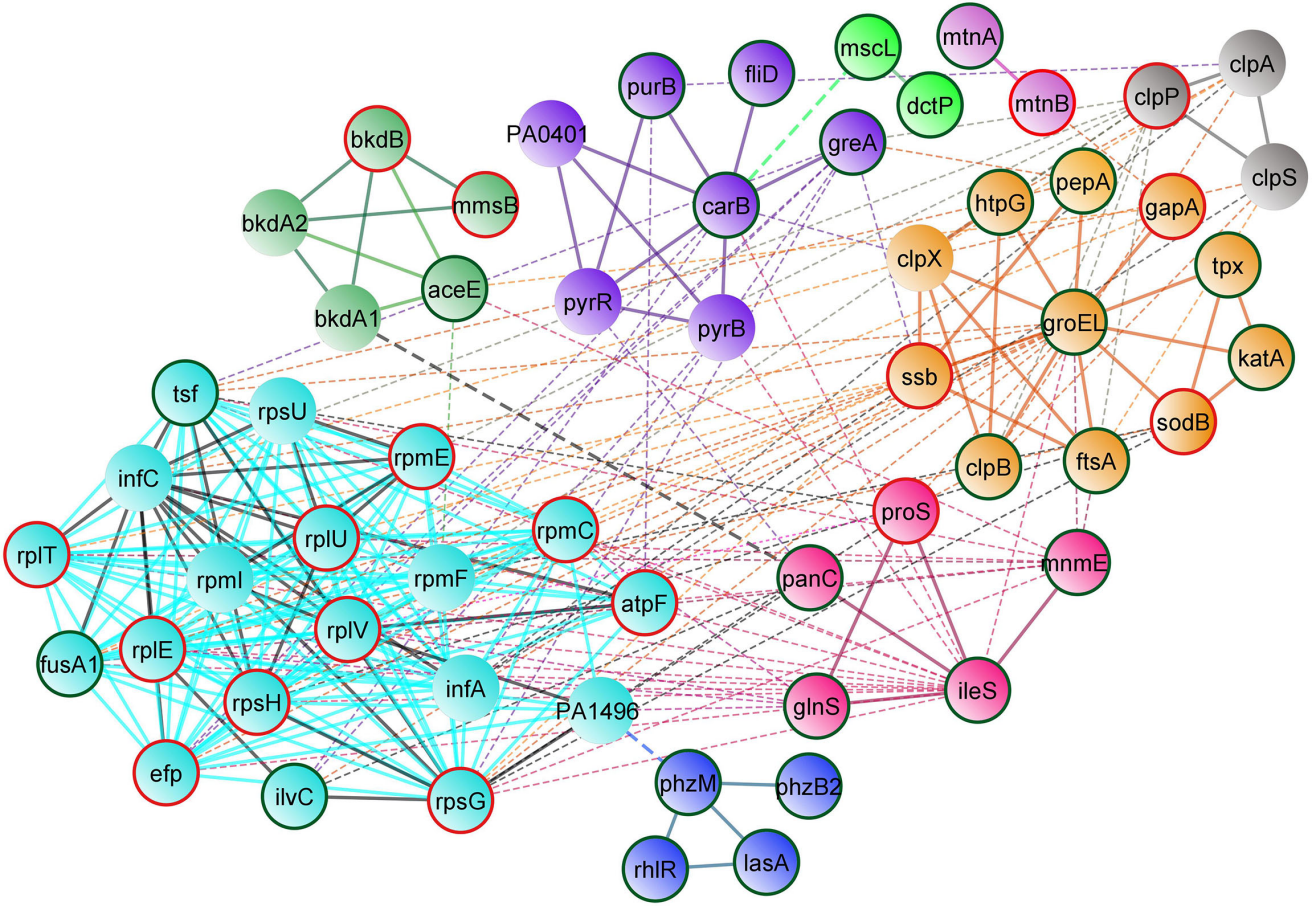
STRING-based protein–protein interaction network of differentially regulated proteins (Interaction score: 0.400 confidence; Clustering: k means algorithm). Physical and functional interaction network of both upregulated and downregulated proteins of UMB-treated cells. Nodes highlighted in red and green represent the upregulated and downregulated proteins, respectively.

Protein–protein interaction network analysis was done using STRING database for both upregulated and downregulated proteins, and an interaction network was constructed using Cytoscape (**Figure 5**). The obtained PPI network with 10 different clusters (K-means clustering algorithm) showed that the interacting protein partners are involved in various biological processes, cellular components, and metabolic pathways, which include proteins involved in stress response, phenazine biosynthesis and aminoacyl tRNA synthetase, protein folding and quorum sensing, biosynthesis of secondary metabolites, cellular nitrogen compound metabolism and antioxidant activity, nucleotide biosynthesis, methionine biosynthesis, lipid bilayer porins, and RNA binding proteins.

### Gene enrichment analysis proves the involvement of differentially expressed proteins in biological process, cellular components, and molecular function

**Figure 6:**
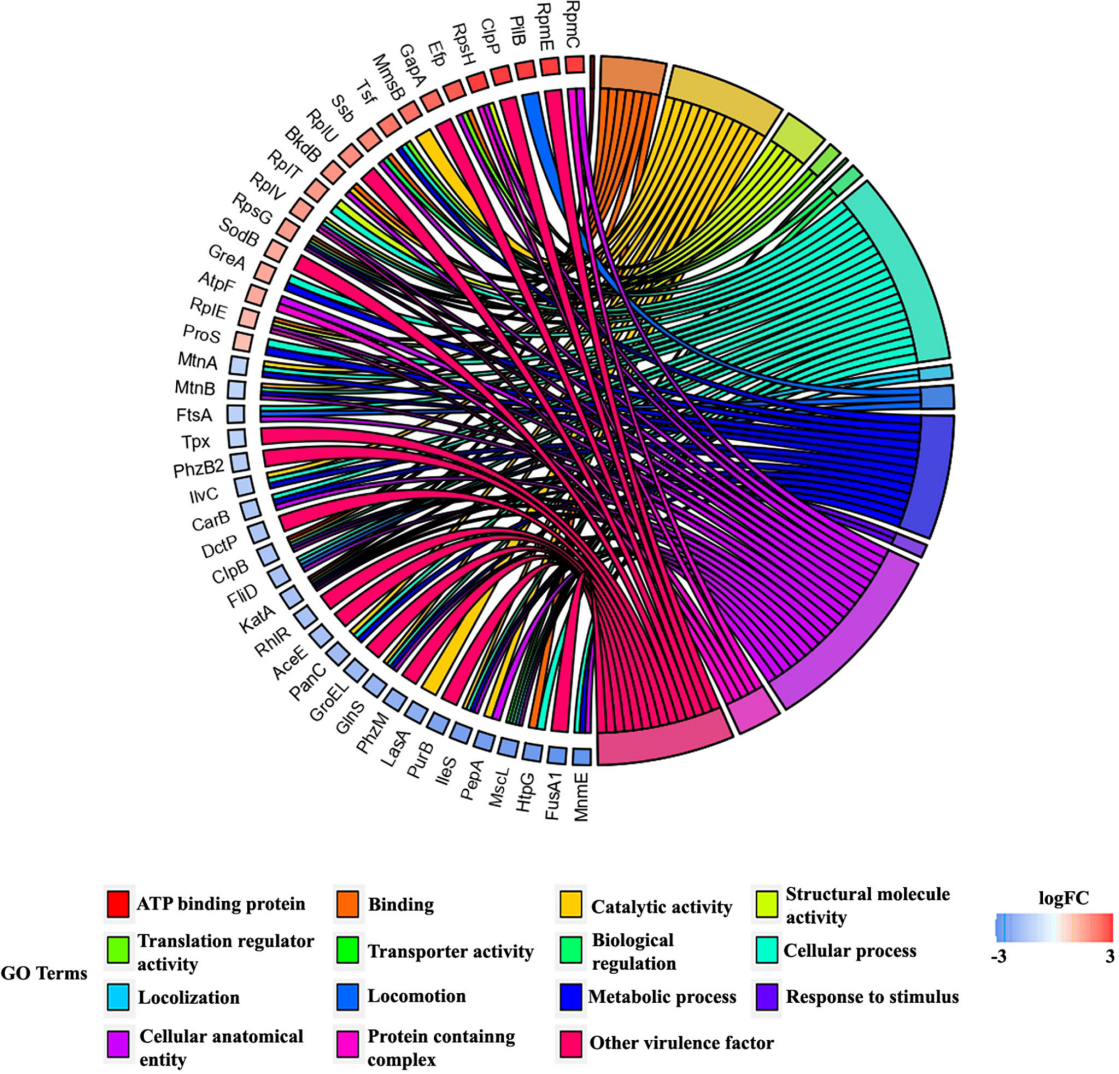
Chord plot representing the GO terms of differentially expressed proteins of treated cells. Each GO term is relevant to a certain biological process and molecular function as given in the legend. The differentially regulated proteins are branched to left hemisphere from the origin of clusters at right hemisphere. Blue and red squares beside protein names at the right hemisphere represent the downregulated and upregulated proteins, respectively (scale bar: logFC −3 to +3). For instance, the downregulated tRNA modification GTPase, MnmE, belongs to the GO terms cellular anatomical entity, metabolic process, and localization. Similarly, the upregulated RpmC (50S ribosomal protein L29) is associated with two GO terms, namely, cellular anatomical entity and response to stimulus.

**Figure 7:**
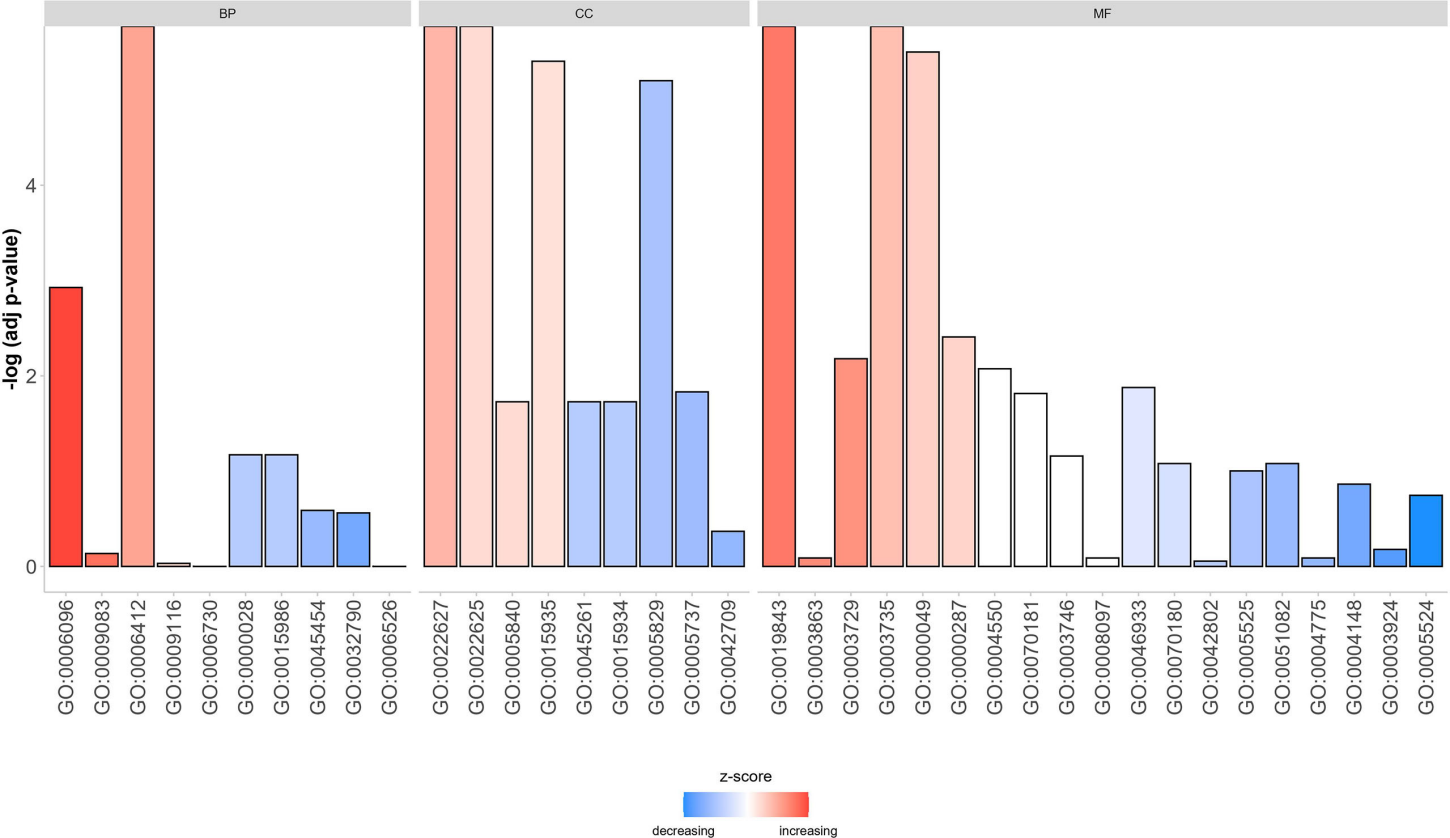
Bar graph representing the cumulative effect of differentially expressed proteins on various GO terms of biological process (BP), cellular component (CC), and molecular function (MF) along with *p*-values and fold change. Here, blue and red bars represent the downregulated and upregulated GO terms, respectively. For example, most of the proteins of GO:0006412 and GO:0015986 are upregulated (represented with red) and downregulated (represented with blue), respectively.

DAVID bioinformatic database-based gene enrichment analysis (GEA) results confirm that the differentially regulated proteins are associated with various functions relevant to the three gene ontology (GO) categories, namely, biological processes, cellular components, and molecular functions in *P. aeruginosa.* The DAVID-based GEA results of both upregulated and downregulated proteins (fold change > ± 1 and *p* ≤ 0.05) are shown in **Figure 6**. Similarly, the GEA results of the individual sets of upregulated and downregulated proteins are shown in **Supplementary Figures 3A, B**, respectively. The distribution pattern of the genes among various clusters confirms that the differentially expressed proteins regulate various functions and processes involved in physiology and virulence factor production of *P. aeruginosa.* Furthermore, based on the functional annotation, differentially expressed proteins were mapped to their enriched GO terms along with *p*-values and fold change to represent the cumulative effect of differentially expressed proteins on various GO terms (**Figure 7**). A total number of downregulated and upregulated proteins present in a particular GO term determines the cumulative expression level of an entire GO term. For instance, the GO term GO:0046933 of molecular function is represented with blue bar denoting that most of the observed proteins belonging to this GO category are downregulated in treated cells of *P. aeruginosa.* Based on this observation, the study confirms that the UMB treatment significantly downregulated the proton-transporting ATP synthase activity and rotational mechanism-associated pathways of *P. aeruginosa.* Similarly, details on the various GO terms and differentially regulated proteins corresponding to particular GO terms are given in **Table 3**.

**Table 3:** Details on the various GO terms and the differentially regulated proteins corresponding to the particular GO terms.

Category	GO Term	Function	Differentially regulated genes in UMB treatment
**Molecular Function**	**GO:0019843**	rRNA binding	*rpsS, rplD, rplE, rplC, rpsM, rplX, rpsH, *rplB, era, rplW, rpsN, rplO, rlmN*, *rplP*, *rpsG, rpsQ*, *rplF*, *rplT*, *rplU, rpsL*, *rpsC, rpsE, rpsD, rplV**
	**GO:0003863**	3-methyl-2-oxobutanoate dehydrogenase (2-methylpropanoyl-transferring) activity	*bkdA1, bkdA2*
	**GO:0003729**	mRNA binding	*rplM, rpsG, *rpsA*, *acnB, rpsC**
	**GO:0003735**	Structural constituent of ribosome	*rpmB,rpsA,rpsB, rplK, rplE, rplX, rpsH, *rplJ, rplQ*, *rpsN, rpmC*, *rplP*, *rplN*, *rplY*, *rpsQ*, *rpsF*, *rplT, rpsC*, *rplV*, *rplS*, *rpsT, rpsI*, *rpsS, rplM, rpsK, rplD*, *rplC, rpsM*, *rplB*, *rplW, rplO, rpsG*, *rpsJ, rplF, rplU*, *rpsD*, *rpsL*, *rpsE, rplR**
	**GO:0000049**	tRNA binding	*metG, thrS, rplE, rpsM, rplP, rpsG, rlmN, rpsJ, rpsL, lysS, pheT, alaS*
	**GO:0000287**	Magnesium ion binding	*purA, bioD, *sucC*, *eno*, *ribB, lysS*, *ppa*, *metK*, *dxs*, *ilvC*, *upp, purF, pheT**
	**GO:0004550**	Nucleoside diphosphate kinase activity	*sucD, sucC, ndk, adk*
	**GO:0070181**	Small ribosomal subunit rRNA binding	*rpsK, era, rpsT, rpsF*
	**GO:0003746**	Translation elongation factor activity	*tsf, fusA, fusB, efp*
	**GO:0008097**	5S rRNA binding	*rplY, rplR*
	**GO:0046933**	Proton-transporting ATP synthase activity, rotational mechanism	*atpG, atpF, atpA, atpH, atpC*
	**GO:0070180**	Large ribosomal subunit rRNA binding	*rplK, rplJ, rplN*
	**GO:0042802**	Identical protein binding	*aspC, azu, aruC, pyrG*
	**GO:0005525**	GTP binding	*folE2, purA, era, fusA, ftsZ, upp, fusB*
	**GO:0051082**	Unfolded protein binding	*groES, clpX, htpG, PA3647, grpE*
	**GO:0004775**	Succinate-CoA ligase (ADP-forming) activity	*sucD, sucC*
	**GO:0004148**	Dihydrolipoyl dehydrogenase activity	*lpdV, lpd, sthA*
	**GO:0003924**	GTPase activity	*era, fusA*, *ftsZ, fusB*
	**GO:0005524**	ATP binding	*clpB, gyrA, groES, atpG, gatB, bioD, xseA, atpA, adk, arcC, alaS, pyrG, metG, thrS, carA, metK, argS, htpG, pheT, sucC, glnA, proS, tyrS, atpC, lysS, clpX, ndk*
**Biological Processes**	**GO:0006096**	Glycolytic process	*lpdV, lpd, gap, aceF, pgi, eno, bkdB, gpmI*
	**GO:0009083**	Branched-chain amino acid catabolic process	*bkdA1, bkdA2, mmsB*
	**GO:0006412**	Translation	*rpmB, gatB, rpsA, rpsB, rplK, rplE, rplX, rpsH, frr, rplJ, rplQ, rpsN, rpmC, rplP, rplN, rplY, rpsQ, rpsF, rplT, rpsC, rplV, rplS, rpsT, rpsI, rpsS, rplM, rpsK, rplD, rplC, rpsM, rplW, rplO, rpsG, rpsJ, rplU, rpsD, rpsL, rpsE, rplR*
	**GO:0009116**	Nucleoside metabolic process	*upp, purF*
	**GO:0006730**	One-carbon metabolic process	*folE2, metK, ahcY*
	**GO:0000028**	Ribosomal small subunit assembly	*rpsS, rpsK, era, rpsG*
	**GO:0015986**	ATP synthesis coupled proton transport	*atpG, atpA, atpH, atpC*
	**GO:0045454**	Cell redox homeostasis	*lpdV, lpd, sthA, trxA, gor*
	**GO:0032790**	Ribosome disassembly	*infC, fusA, fusB*
	**GO:0006526**	Arginine biosynthetic process	*aruC, argJ, carA*
**Cellular Components**	**GO:0022627**	Cytosolic small ribosomal subunit	*rpsS, rpsB, rpsK, rpsH, rpsG, rpsA, rpsQ, rpsF, rpsI, rpsE, rpsC*
	**GO:0022625**	Cytosolic large ribosomal subunit	*rpmB, rplS, rplM, rplK, rplE, rplC, rplX, rplJ, rplB, rplQ, rplW, rplO, rpmC, rplP, rplN, rplY, rplF, rplT, rplR, rplV*
	**GO:0005840**	Ribosome	*rplM, rplD, rpsG, rplU, rpsL*
	**GO:0015935**	Small ribosomal subunit	*rpsM, rpsT, rpsN, rpsJ, rpsI, rpsL, rpsD*
	**GO:0045261**	Proton-transporting ATP synthase complex, catalytic core F(1)	*atpG, atpA, atpH, atpC*
	**GO:0015934**	Large ribosomal subunit	*rplK, rplJ, rplQ, rplV*
	**GO:0005829**	Cytosol	*bioD, hupA, vfr, PSPA7, adk, acnB, ribB, gpmI, ppa, arcC, alaS, pyrG, gcvH2, metG, thrS, nusG, frr, metK, era, gap, dxs, htpG, ilvC, upp, dapF, hemB, aspC, davT, algR, infC, hfq, sucC, rpsT, sthA, grpE, pgi, proS, tyrS, fusB, lysS, ahcY, rpsM, qor, bfr, fusA, hupB, phoP, trxA*
	**GO:0005737**	Cytoplasm	*lpdV, clpB, gyrA, groES, lpd, xseA, adk, efp, gcvH2, tsf, folE2, carA, frr, katA, argS, rlmN, upp, gor, purF, purA, aruC, pepA, phzF1, argJ, glnA, bkdB, clpP1, pyrD, proC, tig, qor, aceF, ftsZ, ndk, trxA, clpP2, fadA*
	**GO:0042709**	Succinate–CoA ligase complex	*sucD, sucC*

Genes highlighted in red and blue represent the upregulation and downregulation of respective proteins in UMB treated cells.

### Phenotypic assay results exemplify the variation on *P. aeruginosa* virulence factor production and activity in UMB treatment

**Figure 8:**
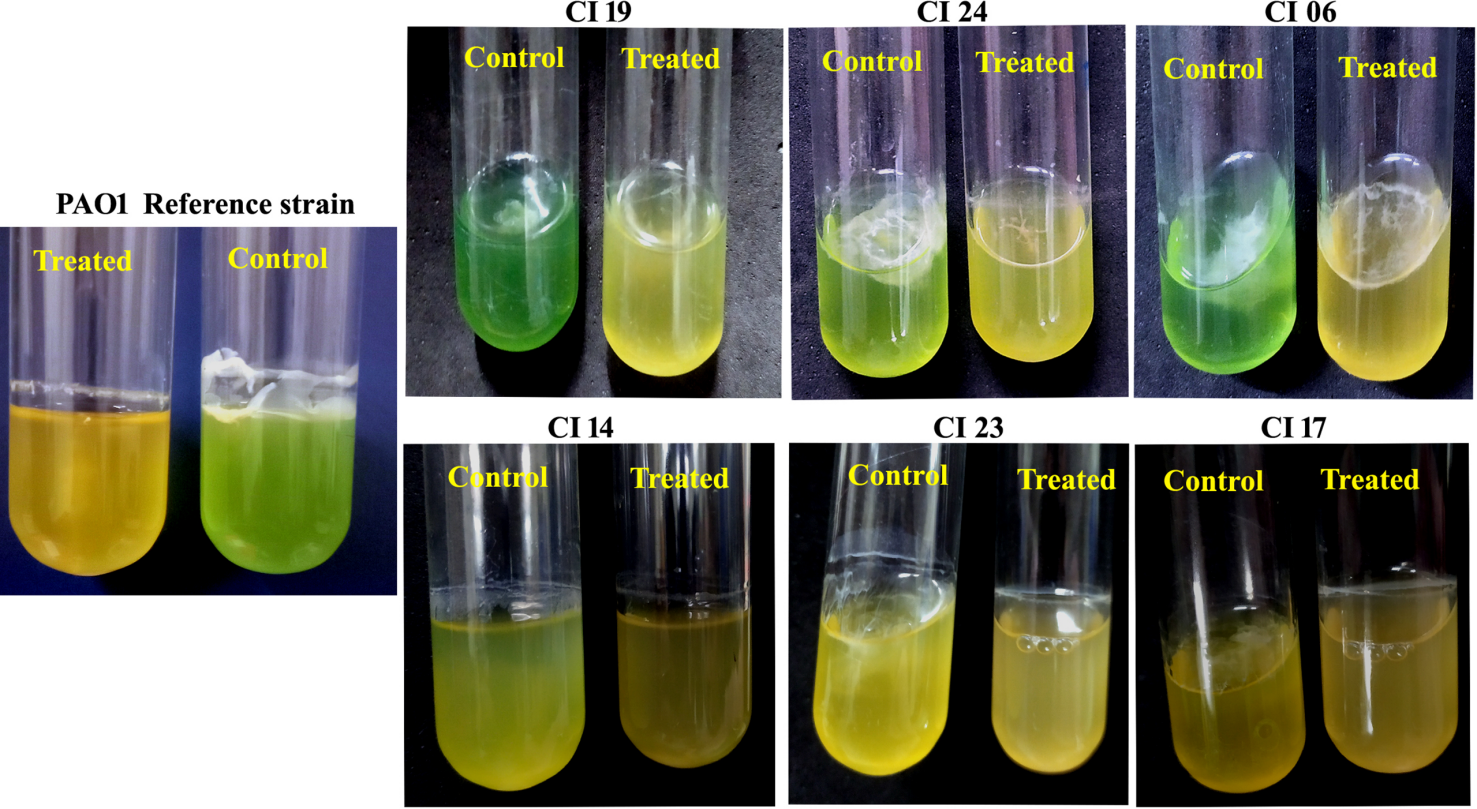
Representative images of 18-h-grown culture of various *P. aeruginosa* strains. Visible reduction could be seen in pyocyanin production and exopolysaccharide formation in treated cells (at MBIC of UMB) when compared to the control cells.

**Figure 9:**
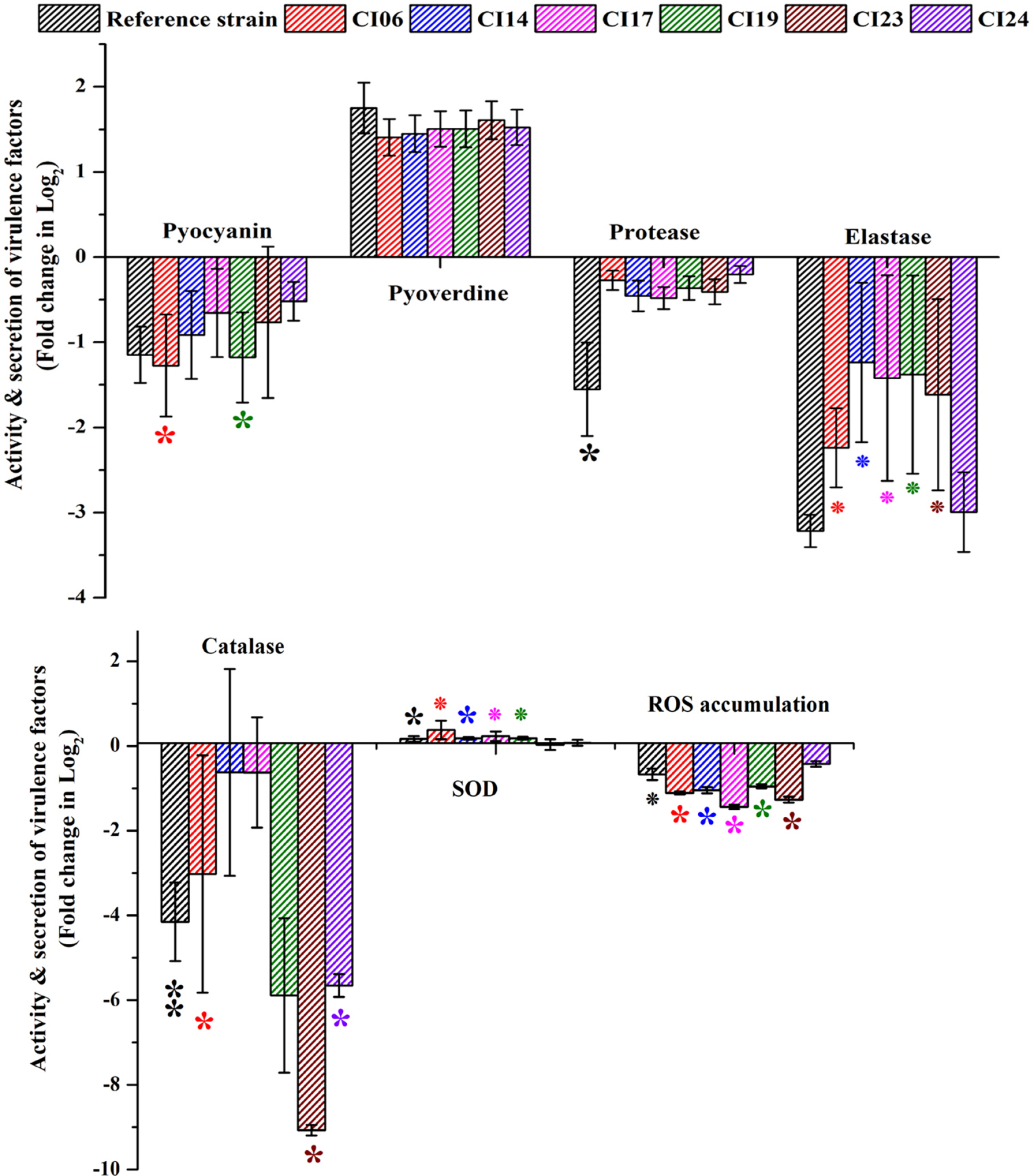
Quantification of various virulence factors of *P. aeruginosa* strains using biochemical assays. ** and * represent significant difference with *p* < 0.01 (*n* = 9) and *p* < 0.05 (*n* = 9), respectively. Increased SOD activity and pyoverdine production and decreased pyocyanin, protease, elastase, catalase, and ROS accumulation were observed in UMB-treated cells.

The impact of UMB on the production of virulence factors such as pyocyanin, pyoverdine, protease, elastase, catalase, and SOD was further analyzed using biochemical assays. The visual observation of 18-h-grown cultures of *P. aeruginosa* reference strain and clinical isolates revealed the reduced level of biofilm formation and pyocyanin production in treated cells compared to the untreated control groups (**Figure 8**). Biochemical quantification results confirm the diminished level of pyocyanin, protease, elastase, catalase, and ROS accumulation and increased level of pyoverdine and SOD in *P. aeruginosa* strains. The observed quantification results were converted to fold change in logarithms (log2FC) and represented as bar graph in **Figure 9**. The antioxidant system-associated enzymes such as catalase and SOD quantification results correlated with the LC-MS/MS expression profile wherein SOD and catalase were found to be upregulated and downregulated, respectively, in UMB treatment. Furthermore, H_2_O_2_ sensitivity assay confirmed the increased susceptibility of UMB-treated cells to the liberated free oxygen radicals on the surface of growth media due to the impaired antioxidant systems in treated cells (**Table 4** and **Supplementary Figure 4**). Moreover, ROS accumulation was found to be significantly reduced in treated cells compared to that in control cells as shown in **Figure 9**. In addition, antibiotic sensitivity assay results showed increased susceptibility of *P. aeruginosa* strains to various antibiotics upon UMB treatment (**Table 5**). Antibiotic sensitivity patterns of the control and treated groups to various drugs are shown in **Supplementary Figure 5**.

**Table 4:** Effect of H_2_O_2_ on *P. aeruginosa* reference strain and clinical isolates in the absence (Control) and presence of UMB (Treated).

	CI06	CI14	CI17	CI19	CI23	CI24	PAO1
**Control**	18.33 ± 0.67	20.11 ± 0.57	18.33 ± 0.67	20.11 ± 0.57	17.44 ± 0.68	16.44 ± 0.68	10.33 ± 0.67
**Treated**	23.67 ± 0.47	23.22 ± 0.79	21 ± 0.82	23.22 ± 0.79	211.22 ± 0.92	22.44 ± 0.49	13.55 ± 0.49

Numerals represent the zone of clearance (mm in diameter ± SD).

**Table 5:** Effect of UMB on antibiotic sensitivity pattern of *P. aeruginosa* strains to various antibiotics.

Antibiotic	CI06		CI14		CI17		CI19	
	Control	Treated	Control	Treated	Control	Treated	Control	Treated
**K**	6.22 ± 3.33	6.22 ± 3.33	10.00	9.33 ± 0.94	7.11 ± 2.51	8.44 ± 0.83	8.44 ± 0.83	10.00
**CTX**	0.00	0.00	17.67 ± 0.67	18.89 ± 0.74	16.00	17 ± 0.67	0.00	0.00
**AK**	24.11 ± 0.31	26.33 ± 0.67	25.78 ± 0.63	26.44 ± 0.68	22.11 ± 1.29	23 ± 0.67	22.67 ± 1.49	25.67 ± 1.25
**CIP**	10.00	14.11 ± 2.6	30.67 ± 1.15	28.33 ± 0.47	23 ± 1.41	29.11 ± 1.37	29.78 ± 0.63	32.44 ± 0.83
**TOB**	24.56 ± 0.50	26.44 ± 0.5	26.56 ± 0.49	26.33 ± 0.67	24 ± 1.49	24.22 ± 1.31	22 ± 0.94	27.33 ± 1.25
**Antibiotic**	**CI23**		**CI24**		**PAO1**			
	**Control**	**Treated**	**Control**	**Treated**	**Control**	**Treated**		
**K**	5.33 ± 3.77	8.00	4.44 ± 3.98	8.89 ± 0.99	10.56 ± 0.50	13 ± 1.15		
**CTX**	16 ± 0.47	17.67 ± 0.67	13.33 ± 1.49	17.33 ± 0.47	14.44 ± 0.50	22.11 ± 0.99		
**AK**	21.78 ± 0.42	23.44 ± 0.50	22.44 ± 0.50	24.56 ± 0.50	23.44 ± 0.50	27.33 ± 1.70		
**CIP**	28.00	30.11 ± 0.31	26.44 ± 0.83	29.56 ± 0.83	21.56 ± 0.50	32.11 ± 0.99		
**TOB**	23.56 ± 0.83	26.44 ± 50	22.00	26.67 ± 1.25	21.78 ± 0.41	28.44 ± 0.50		

Numerals represent the zone of inhibition (mm in diameter ± SD). Kanamycin 30 (K), Cefotaxime 30 (CTX), Amikacin 30 (AK), Ciprofloxacin 5 (CIP), and Tobramycin 10 (TOB).

### qPCR analysis of mRNA transcripts confirms the differential expression of virulence genes

**Figure 10:**
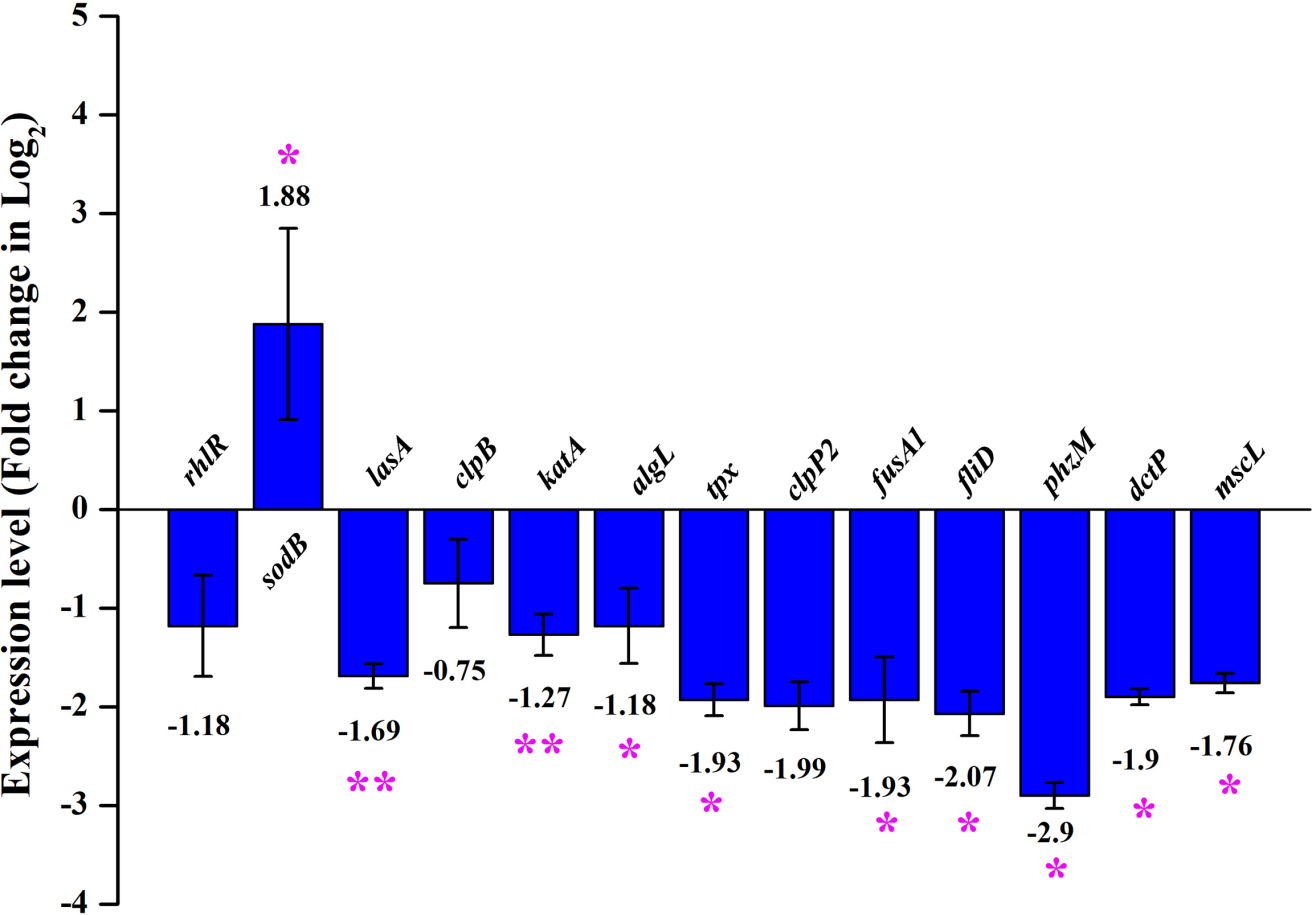
Differential gene expression profile of control and treated (at 125 µg/ml of UMB) cells of *P. aeruginosa* PAO1 in qPCR analysis. Major virulence factors of *P. aeruginosa* are found to be downregulated and *sodB* is observed to be upregulated upon UMB treatment. Error bar represents the standard deviation. * and ** represent the statistical significance between control and treated samples with *p* < 0.05 and *p* < 0.01, respectively (*n* = 6, two experimental and three biological replicates).

The major virulence factors of *P. aeruginosa* were observed to be differentially regulated in UMB-treated cells through mass spectrometric analysis and biochemical assays. The expression profile of differentially regulated proteins and other virulence factors was further validated with qPCR analysis. The obtained gene expression profile evidenced the differential regulation of virulence-related genes in UMB-treated *P. aeruginosa* cells. The virulence genes, *rhlR, lasA, clpB, katA, algL, tpx, clpP2, fusA1, fliD, phzM, dctP*, and *mscL*, were found to be downregulated and *sodB* was upregulated upon UMB treatment (**Figure 10**).

### *In vitro* metabolic viability assay reveals the biocompatibility of UMB on HepG2 cell lines

**Figure 11:**
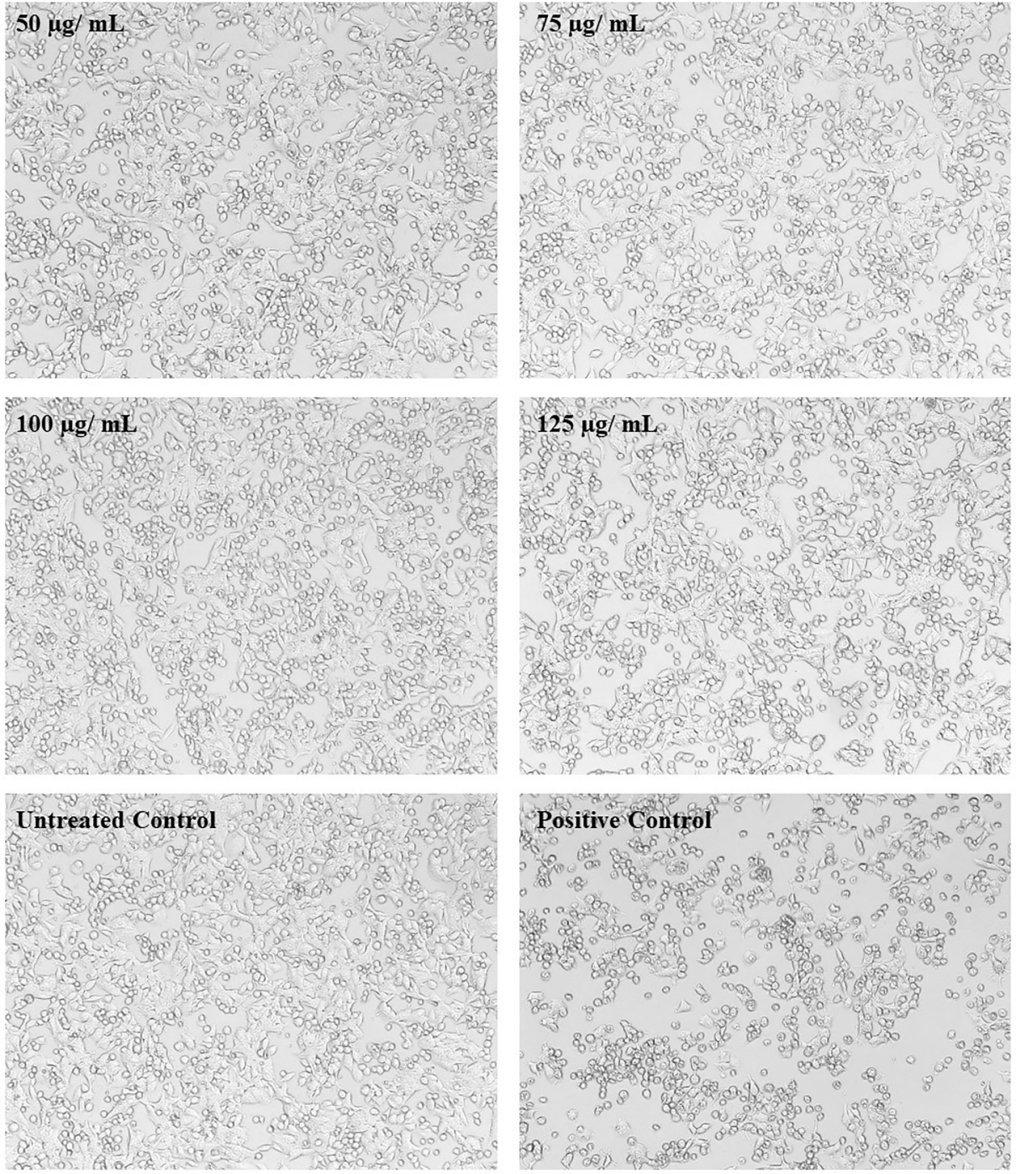
Micrographs showing the effect of different concentrations of UMB on HepG2 cells. The treated cells show no significant change on morphology after 24-h incubation with UMB. Doxorubicin-treated cells (Positive control) show significant variation on the cell morphology (magnification: 10×).

In MTT assay, a yellow tetrazole of tetrazolium salt is converted to a purple formazan product by the NADPH-dependent oxidoreductase activity of active cells of the mitochondrial reaction center, which could be measured calorimetrically. The observed results in the present investigation exemplified the non-toxic nature of UMB to the HepG2 cell lines and the results are given in **Figures 1A, B**. We have observed a minimum of 73.03% cell viability in cells treated with 150 µg/ml of UMB and a maximum of 97.82% viability in the cells treated with 50 µg/ml of UMB. Microscopic observations of treated and untreated HepG2 cells are shown in **Figure 11**. The calculated IC_50_ value of UMB on HepG2 cells is 249.85 µg/ml.

## Discussion

The current investigation aimed to analyze the effect of UMB on the proteome of *P. aeruginosa* through global proteomic profiling using LC-MS/MS analysis. After quantification of differentially regulated proteins between the control and treated groups with mass spectrometric analysis, functional annotation of identified proteins was performed through protein–protein interaction studies and GO analysis. Comparative studies on the proteome of the treated and untreated groups revealed that UMB significantly modulates the expression of noticeable global regulators such as *rhlR*, *lasI, algL, fliD, Tpx*, and *htpG*. These global regulators impair the expression of many virulence genes associated with the pathogenesis and disease establishment traits of *P. aeruginosa.*

Initially, the biofilm inhibitory efficacy of UMB was assessed using *in vitro* assays wherein about 80% inhibition on biofilm formation was observed in *P. aeruginosa* treated with 125 µg/ml of UMB and was determined as MBIC. Metabolic viability results exemplified that the UMB exhibited no adverse effect on bacterial viability at this concentration. Furthermore, growth curve analysis showed no significant alteration in the growth rate of *P. aeruginosa* in both control and treated cells (at 125 µg/ml of UMB). Similarly, the results of enumerating the planktonic and biofilm cells of control and treated (at 15.6 µg/ml to 500 µg/ml) groups showed no altered effect on the growth of planktonic cells while the number of viable cells decreased significantly in the groups treated with 125 µg/ml, 250 µg/ml, and 500 µg/ml of UMB. In addition, the biofilm inhibitory efficacy of UMB in the ring biofilm assay and microscopic analysis revealed the significant antibiofilm efficacy of UMB. *P. aeruginosa* strains form characteristic ring biofilm at the air–liquid interface in the *in vitro* assays (Arai, 2011). In the present investigation, the thickness of biofilm was observed to be decreased at the air–liquid juncture upon treatment with 125 µg/ml of UMB in the ring biofilm assay. Similarly, in microscopic analysis, a firm, thick matrix of biofilm was observed in control groups, while a lower number of dispersed cells were observed in treated groups. In general, various clinical isolates have unique biofilm-forming ability with characteristic strong or weak biofilm formation. Notably, in the present investigation, clinical isolates CI06, CI14, CI23, and CI24 were identified as strong biofilm formers and moderate biofilm formation was observed in CI17 and CI19. Despite the moderate biofilm formation observed in CI19, UMB could impair the biofilm formation of CI19 up to 56.3% ± 0.3%, whereas >80% biofilm inhibition was found in CI06, CI14, and CI24 and >75% inhibition was observed in CI17 and CI24 after treatment with UMB. In addition, the metabolic viability assay on various *P. aeruginosa* strains attested the non-lethal effect of UMB. Considering these observations, the study confirms that the UMB tends to impair the biofilm forming ability of both weak and strong biofilm formers of *P. aeruginosa*. For further insights into the molecular mechanism and primary targets of UMB on *P. aeruginosa*, proteomic profiling was performed using LC/MS-MS analysis.

### UMB modulates the expression of virulence-associated proteins

Functional interaction network analysis of differentially regulated proteins using K means clustering algorithm of the STRING database reveals that the interacting partners of differentially regulated proteins fall into various functional clusters each relevant to the specific virulence and physiology, suggestive of complex molecular mechanisms of UMB on *P. aeruginosa.* For instance, phenazine biosynthesis and QS-related proteins PhzM, PhzB2, RhlR, and LasA interact with proteins involved in translation regulation activity and protein export, which include InfC, Tsf, FusA1, and RplV. The interaction of these two clusters is mediated by the interacting protein partner PA1496. Similarly, the outer membrane proteins MscL and DctP interact with the enzyme CarB, which governs the purine and pyrimidine biosynthesis and biofilm formation in Gram-negative bacteria (Charlier et al., 2018). Hence, visualization of the interacting protein partners of the network analysis highlights the co-regulated proteins and their implications in the global regulatory mechanism of bacterial pathophysiology. Functional interaction of both upregulated and downregulated protein partners within and among the various functional clusters is listed in **Table 6**.

**Table 6:** Interacting partners of differentially regulated proteins of UMB-treated *P. aeruginosa* and their functional clusters.

S. No.	Proteins		Description	Strength	False discovery rate
	Upregulated	Downregulated			
Cluster 1	SodB	PepA, Tpx, KatA	Antioxidant activity and glutathione metabolism	1.59	0.00067
	SodB	KatA, Tpx	Reactive oxygen species metabolic process, and ank repeat	2.34	0.00029
		GroEL, ClpB, HtpG	Stress response and peptidase complex	2.08	6.25e-05
		GroEL, ClpB	Peptidase complex and stress response	2.1	0.00067
Cluster 2	RplT	InfC (IF3), Tsf (EF-Ts)	Mixed, including trans-translation and initiation factor	2.25	0.00016
	RpmC, RplE, RplV, RpsH, RplT, RplU, RpmE, RpsG	FusA1 (EF-G), Tsf	Ribosomal protein	1.86	1.88e-23
	RpmC, RplE, RpsG, RplV, RpsH, RplT, RpmE, RplU	Tsf, FusA1	Ribosomal protein and translation regulator activity	1.84	1.83e-24
	RpmC, RplE, RpsG, RplV, RpsH, RplT, RpmE, RplU	Tsf, FusA1	Ribosomal protein	1.8	1.94e-17
	RplE, RplV, RpsG, RpsH, RplU, RpmE		Ribosomal protein	1.72	7.34e-12
	RplV, RplE		Ribosomal protein	1.67	0.00017
	RplE, RplV, RpsG, RpsH		Ribosomal protein	1.6	8.67e-07
	RpmC, RplE, RpsG, RplV, RpsH, RplT, RpmE, RpsU, RplU, Efp	Tsf, FusA1	Translation and protein export	1.56	3.70e-23
	RpmC, RplE, RpsG, RplV, RpsH, RplT, RpmE, RplU, Efp, AtpF	Tsf, FusA1	Translation and protein export	1.52	3.71e-24
Cluster 3		AlgL	Alginic acid metabolic process	2.84	7.44e-06
Cluster 4		PhzM, PhzB2	Antibiotic biosynthesis and pyridoxamine-phosphate oxidase activity	2.44	0.0066
		RhlR, LasA	Mixed, including beta-ketoacyl-acyl-carrier-protein synthase iii activity and quorum sensing	2.4	0.0066
		PhzM, PhzB2, RhlR, LasA	Phenazine biosynthesis and quorum sensing	2.24	1.90e-06
Cluster 5		CarB	Ribonucleoside monophosphate biosynthetic process	2.22	3.31e-06
		CarB	Ribonucleoside monophosphate biosynthetic process, and 3-carboxymuconate cycloisomerase type II activity	2.14	1.68e-07
		PurB, CarB	Nucleotide biosynthetic process	1.49	0.0020
Cluster 6	BkdB		Dihydrolipoyl dehydrogenase activity and 3-methyl-2-oxobutanoate dehydrogenase activity	2.83	4.36e-06
	BkdB	AceE	Oxidoreductase activity and dihydrolipoyl dehydrogenase activity	2.61	2.20e-07
	BkdB, MmsB	AceE	Mixed, including citrate cycle (TCA cycle) and valine, leucine, and isoleucine biosynthesis	1.76	1.15e-06
Cluster 7	ProS	IleS	Aminoacyl-tRNA editing activity and threonyl-tRNA aminoacylation	2.57	0.0117
	ProS	IleS, GlnS	Aminoacyl-tRNA synthetase	2.18	0.0010
Cluster 8	MtnB	MtnA	L-methionine salvage and spermidine metabolic process	2.75	0.0055
Cluster 9	ClpP (ClpP1)		Mixed, including stress response and peptidase complex	2.35	0.00015
	Other proteins with no significant functional enrichment					
		DctP	C4-dicarboxylate-binding periplasmic protein DctP			
		MscL	Large-conductance mechanosensitive channel			

### UMB impairs the proteins associated with antioxidant activity, reactive oxygen species metabolic process, peptidase complex, and stress response

UMB downregulated the expression of ClpB, GroEL, HtpG, KatA, PepA, and Tpx and upregulated the SodB, which governs the antioxidant activity, stress response, and reactive oxygen species-associated metabolic process. Oxidative stress is one of the major challenges of microbial respiration during normal aerobic metabolism. All biomolecules including DNAs, RNAs, lipids, and proteins are damaged by the range of reactive oxygen species including superoxide ( ${\text{O}}_{2}^{-}$ ), hydrogen peroxide (H_2_O_2_), and hydroxyl radical (OH) (Wan et al., 2019). Furthermore, host immune cell-derived ${\text{O}}_{2}^{-}$ can toxify captured bacteria by damaging the biomolecules on the cell surface. Phagocytic oxidative burst, a defensive mechanism of host system, generates high millimolar H_2_O_2_ in phagosomal vacuole during the event of infection establishment in the host. This host-generated H_2_O_2_ concentration is sufficient to kill the organism. However, *P. aeruginosa* evade the immune response of the host system by employing the free radicals’ scavengers such as catalase (KatA), probable peroxidases (Tpx), and alkyl hydroperoxide reductase (AhpC). Though AhpC enzymes protect the bacteria at lower concentrations of H_2_O_2_, Tpx has a definitive role in protecting the bacteria at sub-millimolar levels of H_2_O_2_, whereas catalase protects *P. aeruginosa* at higher millimolar concentrations of H_2_O_2_ and its role is crucial for the detoxification of endogenous H_2_O_2_. OxyR senses the H_2_O_2_ pool and induces free radical scavenging enzymes, which lower H_2_O_2_ gradient (Sen and Imlay, 2021). In general, SOD catalyzes the conversion of superoxide radicals ( ${\text{O}}_{2}^{-}$ ) to hydrogen peroxide (H_2_O_2_), and the pool of H_2_O_2_ is detoxified by the enzymatic activity of peroxidase and catalase. The absence of peroxidase and catalase increases the accumulation of peroxides and hydroperoxides, which create oxidative stress leading to cell damage. On the contrary, upregulation of superoxide dismutase (SodB) in treated cells might be the indication of bacterial defensive mechanism in eluding the increased stress environment generated by the UMB, whereas downregulation of KatA and Tpx perturbs the cascade mechanism of the antioxidant defense system and leads to the accumulation of intracellular peroxides and hydroperoxides resulting in the oxidative damage in bacteria (**Figure 12.1**). Remarkably, downregulation of the expression of thiol peroxides (Tpx) and catalase (KatA) signifies the diminished antioxidant-mediated tolerance of bacteria, which could enable the pathogen elimination by the host immune system.

**Figure 12:**
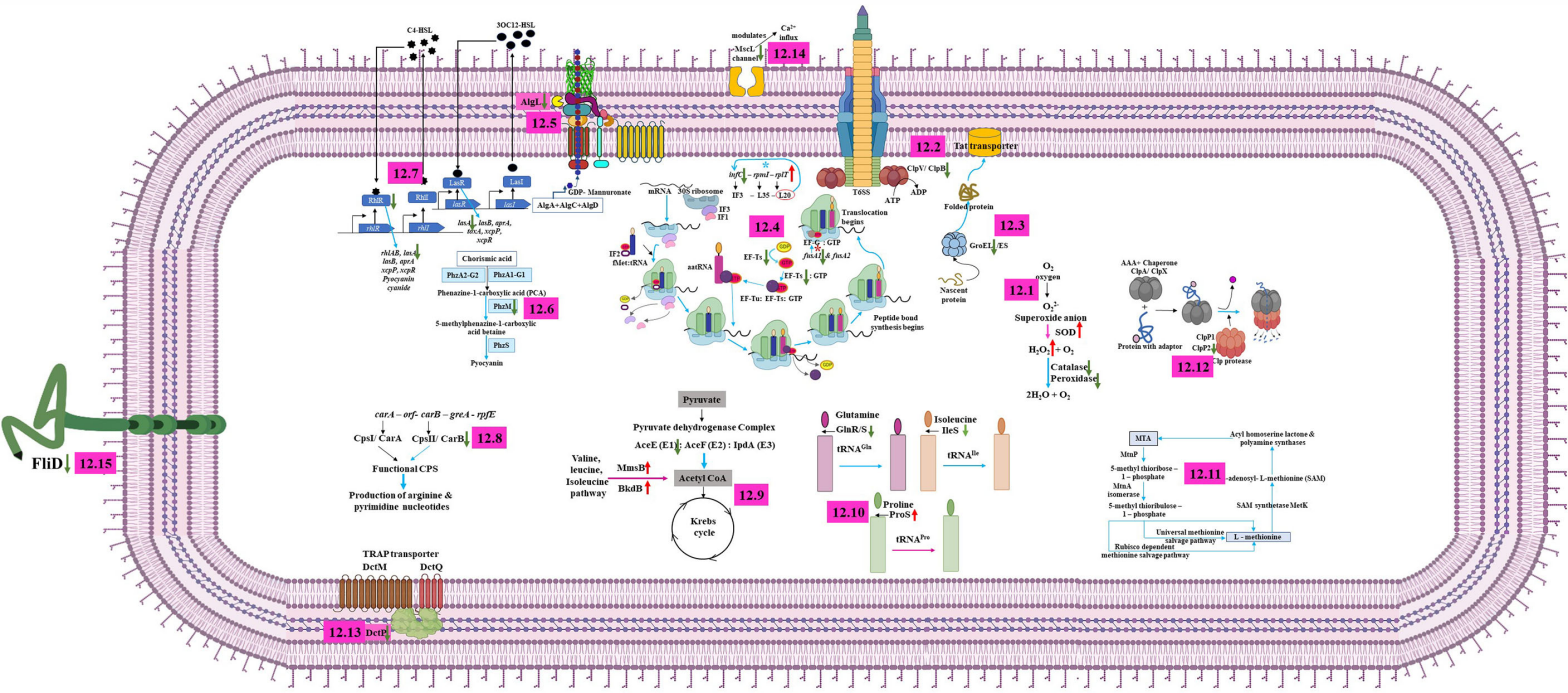
Molecular depicting the mechanism of action of UMB on *P. aeruginosa.* UMB modulates the expression of proteins associated with antioxidant activity, reactive oxygen species metabolic process, peptidase complex, and stress response **(12.1)**; ClpB, which impairs the function of T6SS and plays a vital role in the virulence and phagosomal escape of bacteria **(12.2)**; GroEL, which governs the polypeptides assembly, folding, and transportation of some secretory proteins across the cell membrane and plays a major role in biofilm formation **(12.3)** of *P. aeruginosa*. Ribosomal protein L20, which controls the expression of *infC* operon *via* a transcription attenuation mechanism (Choonee et al., 2007). In this investigation, upregulation of *rplT* (L20) and downregulation of *infC* (IF3), fusA1 (EF-G), and Tsf in treated cells reveal the impact of UMB on ribosomal function, translation, protein export, antibiotic susceptibility, and bacterial virulence **(12.4)**; downregulation of AlgL leads to the accumulation of alginate inside the cell membrane, which destabilizes the bacterial tolerance to the host immune cells and become self-toxic **(12.5)**; UMB downregulates the major virulence factors producing QS systems such as *rhl, las*, and *phzM* of *P. aeruginosa* **(12.6, 12.7)**; UMB differentially regulates the proteins involved in nucleotide biosynthesis pathway **(12.8)**; proteins associated with metabolic process and cellular respiration **(12.9)**; aminoacyl-tRNA synthetase **(12.10)**; 5’-methyl-thioadenosine (MTA) pathway, which maintains the intracellular organic sulfur pool **(12.11)**; ClpP2, which regulates the motility, pigmentation, biofilm formation, and iron scavenging processes **(12.12)**; DctP, which uses an ion electrochemical gradient coupled symporter mechanism for nutrient uptake and is also involved in swarming motility and biofilm formation **(12.13)**; large-conductance mechanosensitive channel (MscL) **(12.14)**; and Flagellar cap protein (FliD), which orchestrates the flagellar assembly on the hook protein and facilitates the polymerization of endogenous flagellin on the growing filaments of flagella **(12.15)**.

Similarly, a molecular chaperone ClpB belonging to a member of class 1 AAA+ proteins of Hsp10/Clp family is reported to be involved in stress response as well as type VI secretion system (T6SS)-mediated virulence mechanisms (Brodmann et al., 2017) (**Figure 12.2**). Downregulation or absence of ClpB impairs the function of T6SS. In addition to that, it plays a vital role in the virulence and phagosomal escape of many pathogenic bacteria including *Listeria monocytogenes*, *Mycoplasma pneumoniae, Porphyromonas gingivalis*, *Mycobacterium tuberculosis*, *Piscirickettsia salmonis*, *Leptospira interrogans*, *Klebsiella pneumoniae*, and *Francisella novicida* (Chastanet et al., 2004; Capestany et al., 2008; Kannan et al., 2008; Estorninho et al., 2010; Lourdault et al., 2011; Isla et al., 2014; Krajewska et al., 2017; Barbosa and Lery, 2019; Alam et al., 2020). Furthermore, among the 20 heat shock proteins, DnaK and GroEL of prokaryotes and GroEL functional homologue HSP60 of eukaryotes are highly conserved and imperative for polypeptides assembly, folding, and transportation of some secretory proteins across the cell membrane (Fujita et al., 1998) (**Figure 12.3**). In addition, GroEL plays a major role in biofilm formation in *Prevotella intermedia* (Karched et al., 2022). Phosphorylated GroEL augments the biofilm formation in *Bacillus anthracis* (Arora et al., 2017). In addition to this, the GroEL paralog GroEL1 modulates the synthesis of mycolic acid, which enables the biofilm formation in *Mycobacterium smegmatis* (Ojha et al., 2005). Furthermore, the *htpG* null mutant of *P. aeruginosa* was observed with impaired motility, decreased biofilm formation, and diminished rhamnolipid and pyocyanin production when compared to the wild-type strains (Grudniak et al., 2018). Similarly, Dong et al. (2021) observed the downregulation of flagellar assembly, infection, and chemotaxis pathway-associated proteins in the *htpG* mutant of *S. Typhimurium* along with attenuated infection establishing phenotypic traits including biofilm formation, motility, invasion, attachment, and inflammation inducement in *in vitro* assays and the BALB/c mice model. Overall, UMB remarkably downregulates the expression of ClpB, GroEL, and HtpG, which confirms that UMB modulates the expression of virulence-associated traits of *P. aeruginosa.*

### Impairment of proteins relevant to ribosomal function, translation, and protein export

In Gram-negative bacteria, the non-ribosomal translation initiation factors, namely, IF1, IF2, and IF3, are key players in protein synthesis. The probable fidelity factor IF3 composed of 180 amino acids governs the assembly of the ternary initiation complex including initiator tRNA, messenger RNA, and the small ribosomal subunit (30S). The ternary initiation complex is an important module in protein synthesis. The 30S ribosomal subunit bound IF3 of the ternary initiation complex alters the equilibrium between 70S ribosomes and their ribosomal subunits (50S and 30S), thus enhancing the availability of free 30S subunits where initiation of protein synthesis begins. The IF3 encoding *infC* is evolutionarily conserved among the Gram-negative bacteria (Butler et al., 1987; McCarthy and Gualerzi, 1990; Liveris et al., 1993). The *infC* operon constitutes *infC-rpmI-rplT* cistrons, which encode IF3-L35-L20, and expression of these three cistrons occurs in specific temporal order and the translation is also interlinked. Lesage et al. (1990) hypothesized the negative regulation of *infC* and *rplT* by the transcription factors IF3 and L20 at the post-transcriptional level in *E. coli.* Later, Pediconi et al. (1995) proved that the downregulation of IF3 induces the L35 expression and the resulting feedback mechanism of L35 enhances the expression of L20 and IF3 in *Bacillus stearothermophilus*. Similarly, Choonee et al. (2007) postulated that the ribosomal protein L20 controls the expression of *infC* operon *via* a transcription attenuation mechanism in *B. subtilis.* Interestingly, in the present investigation, the obtained expression profile of *infC-rpmI-rplT* cistrons was consistent with the previously reported studies wherein InfC (IF3) was downregulated and RplT (L20) was upregulated in UMB-treated *P. aeruginosa* cells. These observed results suggest that UMB modulated the expression of ribosomal proteins associated with the cellular functions of *P. aeruginosa* (**Figure 12.4**).

In addition, Elongation factor Tu (EF-Tu), Elongation factor Ts (EF-Ts), and Elongation factor G (EF-G) are major factors in the translation elongation process of bacteria. EF-Ts (Tsf, stable component) is essential for EF-Tu (Tuf, unstable component)-GTP-aa-tRNA ternary complex formation wherein Tsf enhances the interaction of Tuf with GDP/GTP by acting as a guanosine diphosphate and guanosine triphosphate exchanger (Lucas-Lenard and Lipmann, 1966). Post-translational modifications on Tuf impair the bacterial virulence, dormancy, stress tolerance, and sporogenesis (Talavera et al., 2018). Thus, downregulation of Tsf upon UMB treatment perturbs the virulence trait of *P. aeruginosa* by impairing the EF-Tu-GTP-Ef-Ts complex. Furthermore, *fusA1* and *fusA2* encode EF-G in *P. aeruginosa* (**Figure 12.4**). A previous study reported the gentamycin-, tobramycin-, and amikacin-resistant phenotypic traits of the strains containing mutation at EF-G1A domains II, III, and V while mutation at domains IV and V enhanced the aminoglycoside susceptibility of *P. aeruginosa* strains (Bolard et al., 2018). However, none of the previous studies explicitly commented on the *fusA1* null mutant because it is an essential gene for bacterial physiology. In addition to the above discussions, downregulation of FusA1 was observed in UMB-treated *P. aeruginosa* strains, and further, the treated cells were found with increased susceptibility to the aminoglycosides such as amikacin, tobramycin, and kanamycin. Moreover, the UMB-treated cells showed increased susceptibility to ciprofloxacin and cefotaxime when compared to the untreated control groups. Taken together, observed results revealed that UMB had curbed the key factors related to the elongation factor, protein synthesis, and stress tolerance.

### Protein(s) of the alginic acid metabolic process

Extracellular polysaccharide is a major component of bacterial biofilm matrix and many species adept at biofilm formation secrete a cluster of polysaccharides. In *P. aeruginosa*, alginate, Pel, and Psl exopolysaccharides aggregate on the airways of CF lung and bacteria residing inside the exopolysaccharide matrix increase the antimicrobial tolerance, which culminates in the development of chronic infection (Jennings et al., 2021). The mucinous exopolysaccharide alginate evades the host immune mechanism and jeopardizes patient recovery (Jain and Ohman, 2005). The anionic polysaccharide alginate is composed of sugar monomers mannuronic acid and guluronic acid. The proteins for alginate biosynthesis are encoded by the 12-gene *algD* operon. The *algL* is a notable gene in the alginate biosynthesis gene cluster, which encodes homeostasis enzyme alginate lyase (Schiller et al., 1993). AlgL cleaves the alginate polysaccharide and clears the periplasmic space of bacterial cells, whereas the Δ*algL* mutant fails to export the accumulated alginate from periplasmic space (**Figure 12.5**). The alginate accretion inside the cell membrane disintegrates the outer membrane, which destabilizes the bacterial tolerance to the host immune cells and becomes self-toxic (Bakkevig et al., 2005; Gheorghita et al., 2022). Interestingly, in this investigation, downregulated AlgL could increase the accumulation of intracellular polysaccharide, resulting in cell membrane disintegration. Thus, the study confirms the anti-virulent efficacy of UMB against *P. aeruginosa* that can potentially challenge the bacterial adaptation in the host environment.

### Regulatory proteins of phenazine biosynthesis and quorum sensing

In the host pathogen arms race, bacteria rapidly evolve to the changing environments of the host system by the alarming signal of the quorum sensing (QS) mechanism. QS is the density-dependent cell–cell communication of the bacteria wherein the hierarchy of signaling molecules controls the expression of biofilm formation and virulence-associated genes (Moura-Alves et al., 2019). Hence, anti-QS molecules can help the host immune system to counteract some of the detrimental effects of virulence and biofilm-mediated damage. In *P. aeruginosa*, the two operons, namely, *phzA1B1C1D1E1F1G1* (*phz1* operon) and *phzA2B2C2D2E2F2G2* (*phz2* operon), synthesize the functional phenazine. The deletion mutant of *phz1* operon overexpressed the *phz2* operon as a homeostasis response (Cui et al., 2016). Literature survey shows that any one of the phenazine operons is sufficient for synthesis of the pyocyanin precursor, phenazine-1-carboxylic acid (PCA). PCA is subsequently converted to pyocyanin by the phenazine-modifying enzymes, phenazine-specific methyltransferase (PhzM) and flavin-containing monooxygenase (PhzS). Notably, insertional inactivation of phzM or phzS exhibited the pyocyanin-deficient phenotypes of *P. aeruginosa* (Mavrodi et al., 2001) (**Figure 12.6**). Generally, pyocyanin is a redox-active pigment and has an important role in disease establishment in airway epithelial cells of the respiratory tract by interfering the host cell electron transport, cellular respiration, and energy metabolism, and it also sequesters the irons from the host system during pathogenesis (Rada and Leto, 2013). In the current investigation, LC-MS/MS results showed the downregulation of PhzB2 and PhzM in UMB-treated *P. aeruginosa* when compared to the untreated control groups. These observations are similar to the previous studies that elaborated the function of *phzB2* and *phzM* genes in the biosynthesis of pyocyanin that are important virulence factors of *P. aeruginosa.*

In addition to this, it is well known that the two QS systems *rhl* and *las* of *P. aeruginosa* regulate the expression of many virulence gene products during pathogenesis. The two canonical LuxI/R-type QS systems, RhlI/R and LasI/R, produce and bind to the cognate autoinducers butyryl homoserine lactone (C4-HSL) and 3-oxo-dodecanoyl homoserine lactone (3O-C12-HSL), respectively, and activate the expression of biofilm formation and other virulence-associated gene cascades (Medina et al., 2003). Mukherjee et al. (2017) have shown that the Δ*rhlR* mutant results in attenuated virulence traits during animal infections whereas the Δ*rhlI* mutant retains the virulence traits. Similar to that, we also observed the downregulation of the *rhlR* expression in treated *P. aeruginosa* cells. Three exoproteases, namely, LasA, elastase, and alkaline proteases, are other important virulence traits of pathogenic *P. aeruginosa* strains that enhance the microbial tolerance to the host environment (Coin et al., 1997). Expression of these proteases is under the control of *lasR* and *rhlR* QS systems (Toder et al., 1994) (**Figure 12.7**). In this investigation, we have observed that UMB treatment downregulates the expression of LasA proteases. Taken together, downregulation of PhzB2, PhzM, RhlR, and LasA in treated cells exemplified the potential anti-QS efficacy of UMB.

### Proteins involved in the nucleotide biosynthetic process

In this study, the enzymes involved in purine and pyrimidine biosynthesis such as CarB and PurB were found to be downregulated in UMB-treated *P. aeruginosa* cells. Previous studies revealed that these two enzymes are also attributed to the virulence traits of *P. aeruginosa*. For instance, carbamoylphosphate synthetase (CPS) is a heterodimeric (αβ)_4_ protein that is encoded by *carAB* operon consisting of the *carA-orf-carB-greA-rpfE* gene cassette. The CPS catalyzes the synthesis of carbamoylphosphate (CP) from bicarbonate, ATP, and glutamine. CP is a precursor for arginine and pyrimidine biosynthesis pathway and regulates the *de novo* synthesis of both arginine and pyrimidines. CPS is composed of a small, amidotransferase subunit CPS I and a large synthetase subunit CPS II that are encoded by *carA* and *carB*, respectively (**Figure 12.8**). The Δ*carB* mutant reduced the swimming motility on semi-solid agar and also decreased biofilm formation on polystyrene surfaces in *Xanthomonas citri* subsp. *citri* (Zhuo et al., 2015; Charlier et al., 2018). Similarly, adenylosuccinate lyase (ASL, known as PurB), an enzyme involved in purine biosynthesis *via* both salvage pathway and *de novo* pathway, has been recognized as a potential drug target against microbial infections (Banerjee et al., 2014).

### Proteins associated with metabolic process and cellular respiration

Generally, during the process of energy production in a cell, the pyruvate dehydrogenase complex converts the pyruvate into acetyl-CoA, which is then used as a substrate in the citric acid cycle/glyoxylate cycles and fatty acid synthesis pathway. The pyruvate dehydrogenase complex contains a pyruvate dehydrogenase E1 component (AceE), dihydrolipoyllysine-residue acetyltransferase (AceF), and dihydrolipoyl dehydrogenase (IpdA). In the present study, we have observed the reduced expression of AceE, which indicates that UMB has impaired the pyruvate-to-acetyl CoA conversion, which could perturb the TCA cycle, whereas the 3-hydroxyisobutyrate dehydrogenase (MmsB) and lipoamide acyltransferase component of the branched-chain alpha-keto acid dehydrogenase complex (BkdB) was found to be upregulated. MmsB and BkdB produce acetyl CoA through valine, leucine, and isoleucine pathways and thus act as adaptive mechanisms to equilibrate the cellular energy production; they support microbial adaptation to the harsh environment (**Figure 12.9**) (KEGG reference pathway).

### Aminoacyl-tRNA synthetase and threonyl-tRNA aminoacylation

*glnS* synthesizes an aminoacyl tRNA synthetase, glutaminyl tRNA-synthetase (GlnRS), which attaches to its cognate amino acid glutamine during protein synthesis. GlnRS is not found in all organisms, and some prokaryotes also lack GlnRS. In the absence of GlnRS, glutamine is attached to glutamate tRNA-synthetase (GluRS), which is later converted to GlnRS by amidation upon binding to the tRNA^Gln^ and becomes GlnRS-tRNA^Gln^ (Akochy et al., 2004), whereas in *P. aeruginosa*, GluRS discriminates its cognate amino acid (glutamate) and thus fails to attach to non-cognate glutamine, which demands the formation of functional GlnRS (Hu et al., 2015; Escamilla et al., 2020). In the present study, UMB specifically downregulated the expression of *glnS* that could affect the protein synthesis in *P. aeruginosa.* Similarly, *proS* and *ileS* encode proline tRNA ligase and isoleucine tRNA ligase, respectively. ProS catalyzes the attachment of proline to tRNA^Pro^ and IleS catalyzes the attachment of isoleucine to tRNA^Ile^. In the present study, IleS was found to be downregulated, whereas ProS was upregulated in UMB-treated cells (**Figure 12.10**). Thus, the study confirms that UMB modulates the cellular function of *P. aeruginosa* at the MBIC concentration.

### L-methionine salvage and spermidine metabolic process

Maintaining the intracellular organic sulfur pool is an essential metabolic process in all living cells (Mary et al., 2012). For instance, sulfur is essential for the synthesis of L-cysteine and L-methionine (S-containing amino acids), thiamine and coenzyme A (coenzymes), and glutathione and S-adenosyl-L-methionine (SAM) (co-factors) (Miller et al., 2018). SAM serves as a co-factor for numerous enzymes and acts as a methyl donor for vital macromolecules including DNA, RNA, lipids, and proteins (North et al., 2020). Furthermore, SAM donates the homoserine lactone for acyl and aryl homoserine lactones during the biosynthesis of QS molecules. The SAM derivative, 5’-methyl-thioadenosine (MTA), is an intermediate metabolite of the methionine salvage pathway. Intracellular accumulation of MTA induces cell cytotoxicity and, hence, MTA is recycled to L-methionine, L-adenine, and formate through the canonical pathway, which consists of six successive enzymatic reactions during aerobic metabolism in *E. coli*. A group of six enzymes are essential for the cascade reactions, which include nucleosidase/phosphorylase (MtnP), isomerase (MtnA), dehydratase (MtnB), enolase/phosphatase (MtnC), dioxygenase (MtnD), and transaminase (MtnE) (Albers, 2009), whereas during anaerobiosis in *R. palustris* and *R. rubrum*, MTA is metabolized into ethylene *via* a dihydroxyacetone phosphate-methanethiol shunt (DHAP-ethylene shunt) by the enzymatic activity of MtnP and MtnA (Miller et al., 2018). These previous studies exemplify the importance of MtnA in recycling the toxic intracellular MTA metabolite. In this present investigation, we have observed the downregulation of MtnA, whereas MtnB was found to be upregulated (**Figure 12.11**). Since the activity of all the enzymes are mandatory for this MTA recycling pathway, perturbation of MtnA expression upon UMB treatment might have interrupted the process that could lead to altered SAM synthesis, resulting in the depletion of SAM for biosynthesis of QS molecules.

### Mixed, including stress response and peptidase complex

Caseinolytic peptidases (ClpPs) are ATP-dependent proteolytic machineries belonging to the AAA+ protease family. ClpPs regulate the intracellular protein degradation that is essential for bacterial growth and cell division. The ClpPs contain the proteolytic heptameric barrel ClpP and hexameric ATPases ClpA and ClpX in both *E. coli and P. aeruginosa* (Zhao et al., 2016). ClpP contains two isoforms, namely, ClpP1 and ClpP2, and these proteins regulate the motility, pigmentation, biofilm formation, and iron scavenging processes in *P. aeruginosa.* In particular, ClpP2 was found to be important in the regulation of the virulence traits, especially microcolony formation during the initial stage of biofilm formation (Hall et al., 2017; Mawla et al., 2021). Moreover, Zhao et al. (2016) have shown that the mice infected with Δ*clpS*Δ*clpA* and Δ*clpP* survived longer (85%–100% after 96 h) than the mice infected with the wild type (70% death after 80 h). Personne et al. (2013) have experimentally proved that ClpP1 and ClpP2 are co-transcribed in *M. tuberculosis.* Of particular interest, we have observed the downregulation of ClpP2 (1.26-fold, UniProt ID: Q9HYR9) and the upregulation of ClpP1 (3.2-fold, UniProt ID: Q9I2U1) in UMB-treated cells of *P. aeruginosa.* This prompts us to speculate that ClpP1 and ClpP2 might be transcribed under the control of two different promoters unlike *M. tuberculosis.* Interestingly, similar to the present investigation, Hall et al. (2017) have shown that the ClpP1 operon (PA1801) is distinct to the ClpP2 (PA3326) operon, whereas the ClpP1 operon shares the regulatory mechanism with the ClpX expression in *P. aeruginosa* (**Figure 12.12**). Collectively, mass spectrometric analysis unravels the differential expression of major virulence factors of *P. aeruginosa* in UMB-treated cells.

### Other significantly altered virulence-associated proteins without functional enrichment score in STRING interaction analysis

#### C4-dicarboxylate-binding periplasmic protein DctP

In order to uptake the nutrients from a competitive niche, bacteria and archaea have evolved with various nutrient uptake systems called transporters. The prevalent solute-binding protein-dependent nutrient uptake transporters are ABC transporter, TRAP transporter, and secondary transporter. TRAP transporters contain a tripartite ATP-independent solute-binding subunit called DctP (Mulligan et al., 2011), which uses ion electrochemical gradients coupled symporter mechanism for nutrient uptake (Rosa et al., 2018). In *Vibrio alginolyticus*, the Δ*dctP* mutant was observed with reduced swarming motility, biofilm formation, and cell adhesion compared to the wild type (Zhang et al., 2022). In line with a previous report, we have also observed UMB downregulating the expression of DctP (fold change: −1.57; *p*-value: 0.004) in the treated group when compared to the control group (**Figure 12.13**). Furthermore, qPCR analysis also showed the downregulation of dctP in UMB-treated cells with −1.9-fold change (*p*-value: 0.003). Thus, it is apparent that UMB attenuates the multiple virulence factors of *P. aeruginosa* by targeting the numerous global regulators involved in virulence production.

#### Large-conductance mechanosensitive channel

Gram-negative bacteria have numerous protein export systems, for instance, types I, III, and IV secrete proteins across the dual membrane of bacterial envelope, whereas Sec, Tat, MscL, and Holins secrete proteins across the inner membrane (Ma et al., 2003). MscL is an essential channel and opens in response to membrane tension in hypoosmotic environment and accounts for the rapid release of solutes into the host system, thus preventing the bacteria from osmotic down-shock (Ajouz et al., 1998; Wahome and Setlow, 2008). Guragain et al. (2013) have shown that Ca^2+^ induced MscL valve, limiting the Ca^2+^ influx, whereas MscL-deprived cells become more accessible to Ca^2+^ influx. In addition, increased permeability of Ca^2+^ inside the cells has been shown to augment the swimming motility in *V. parahaemolyticus* (Gode-Potratz et al., 2010). On the contrary, Ca^2+^ influx has been shown to attenuate the motility in fluorescent pseudomonads (Sakai et al., 2003). Since the experimental information on Ca^2+^ influx and MscL channel function in *P. aeruginosa* are obscure, more detailed studies are required for defining the role of MscL in *Pseudomonas.* However, in this study, UMB treatment reduced the expression of MscL channels (proteomic profiling: fold change −2.72, *p*-value: 0.02; qPCR analysis: fold change −1.76, *p*-value: 0.04), and based on the previous reports, it could be hypothesized that UMB curbs the protective role of the MscL channel against osmotic swelling and perturbs the adaptive mechanism of *P. aeruginosa* to the extreme down-shocks. On the other hand, some previous studies have shown the increased Ca^2+^ influx and enhanced swimming motility in cells with perturbed MscL. Since it has already been shown that UMB could downregulate many QS and virulence-associated genes, in this study, we speculate that UMB could have affected the MscL-mediated osmotic balance of *P. aeruginosa* by downregulating the MscL (**Figure 12.14**). Additionally, the filament-cap gene (*fliD*) was also found to be downregulated upon UMB treatment, which orchestrates the flagellar assembly on the hook protein and facilitates the polymerization of endogenous flagellin on the growing filaments of flagella (Macnab, 1992; Yokoseki et al., 1995) (**Figure 12.15**). The major pilin proteins PilB and PilA are involved in bacterial adhesion and biofilm formation (Bogiel et al., 2021). Besides PilB (3.2-fold with *p*-value 0.01) being upregulated, PilA (1.35-fold with *p*-value 0.08) was found to be downregulated in UMB treatment, which impairs the expression of functional pilin proteins. Hence, the study confirms that UMB significantly affects the factors involved in motility, attachment, and biofilm formation of *P. aeruginosa.*

Furthermore, the KEGG pathway enriched genes and GO classification of differentially regulated proteins exemplify that the differentially regulated proteins are associated with various GO terms in functions relevant to ATP binding, binding, catalytic activity, structural molecule activity, translation regulator activity, transporter activity, biological regulation, cellular process, localization, locomotion, metabolic process, response to stimulus, cellular anatomical entity, protein-containing complex, and other virulence functions as given in the chord plot (**Figure 6**). In addition, the bar graph (**Figure 7**) results reveal that the differentially expressed genes are part of various GO terms with functional implications in biological process, molecular function, and cellular components. Each GO term represents the cluster of proteins involved in the closely related biological activity. The upregulated and downregulated GO terms are highlighted in red and blue bars, respectively. The upregulated GO terms contain a higher number of upregulated proteins, and *vice versa*, in the corresponding GO term. For example, the GO term GO:0051082 of molecular function contains *groES, clpX, htpG*, PA3647, and *grpE* genes that regulate protein folding and binding. Among these genes, *groES, clpX, htpG*, and *grpE* were upregulated and PA3647 was downregulated, and hence, it is said that the entire GO term is downregulated. Similarly, the GO term GO:0003735 containing proteins with a function relevant to the structural constituent of ribosome is considered to be upregulated as most of the proteins of the GO term are upregulated.

The quantification of differentially expressed virulence factors of *P. aeruginosa* through biochemical assays and qPCR analysis is comparable to the LC-MS/MS proteomic profiling. Biochemical analysis results showed the presence of the high concentration of pyoverdine and the decreased level of pyocyanin, protease, and elastase in UMB-treated cells. Little et al. (2018) demonstrated the overexpression of pyoverdine and downregulation of pyocyanin in *algR D54E* mutants and carbon source influenced the pyoverdine expression than the extracellular iron concentration. Furthermore, *algR* regulates the expression of a 12-gene operon composed of *algD, alg8, alg44, algK, algE, algG, algX, algL, algI, algF*, and *algA* encoding proteins associated with alginate biosynthesis, modification, and export (Okkotsu et al., 2014). In the present investigation, *algL* expression was found to be downregulated in both proteomic profiling (fold change: −1.90, *p*-value: 0.01) and transcript analysis (fold change: −1.18, *p*-value: <0.05). Similarly, biochemical quantification results showed the decreased pyocyanin and increased pyoverdine production. Together, these interpretations support the notion that pyoverdine might increase as the bacterial survival mechanism circumvents the iron-limiting condition and available carbon source in an environment, which is comparable to the observation made by Sethupathy et al. (2016) wherein curcumin-treated *P. aeruginosa* cells have shown similar phenotypic traits. Furthermore, elastase and LasA protease are reported to be representative of many secreted proteases (Kessler et al., 1998). Secretion of elastase A (LasA, staphylolysin) and elastase B (LasB) is regulated by the QS system of *P. aeruginosa.* LasB degrades elastin, collagen, fibronectin, mucin, and vascular endothelial cadherin of cellular junctions, leading to tissue injury and bacterial dissemination (Cigana et al., 2021). Furthermore, elastase digests the thrombin and releases thrombin-derived peptide FYT21, which hijacks the host pro-inflammatory responses (Van Der Plas et al., 2016). Moreover, LasB interferes alveolar macrophages and inactivates the C3 complement system, which aids in the establishment of chronic lung infection especially in CF patients (Scott et al., 2013; Cigana et al., 2021). In the present investigation, protease and elastase production was found to be decreased in the biochemical analysis of the control and treated groups, similar to the results observed in proteomic profiling and qPCR analysis. Collectively, UMB downregulates the expression of alginate, elastase, protease, and pyocyanin, which are considered to be part of major virulence factors of the *P. aeruginosa* secretome in airway epithelial cells and the lung surface of CF patients. Furthermore, the concentration of SOD was found to be increased in phenotypic assays and expression analysis (proteomic profiling: FC −1.49; qPCR: FC −1.88, *p*-value < 0.5). Later, increased H_2_O_2_ susceptibility and decreased catalase production were observed in the treated cells of various *P. aeruginosa* clinical isolates. Conceivably, *katA* (catalase) expression was 1.27-fold and 1.65-fold (*p*-value = 0.01) downregulated in qPCR and mass spectrometric analysis, respectively. The unique catalase of *P. aeruginosa* is highly stable, is extracellularly present, and elicits the peroxide resistance in planktonic and biofilm cells of *P. aeruginosa* (Shin et al., 2008). This suggests that the altered catalase expression in UMB treatment impairs the conversion of H_2_O_2_ into H_2_O and O_2_. Despite the decreased catalase production, upregulated SOD might rescue the organism from free oxygen radicals of the environment. Notably, ROS accumulation was found to be decreased in treated cells, which enumerates the antioxidant property of UMB. Previous studies also reviewed and reported the antioxidant properties of UMB in higher animal models (Singh et al., 2010; Sim et al., 2015; Mazimba, 2017). The observed biochemical assay results of various *P. aeruginosa* clinical isolates seem to be similar to that of results obtained for the reference strain. Hence, the study supports the conclusion that UMB perturbs the virulence factors of a broad range of *P. aeruginosa* strains.

In addition to this, UMB increased the susceptibility of treated cells to various antibiotics including kanamycin, ciprofloxacin, amikacin, tobramycin, gentamycin, and cefotaxime. The clinical isolates were observed with varying phenotypic traits against antibiotics in the antibiogram. Increased AK30 susceptibility was observed in all the test strains treated with the MBIC concentration of UMB. In tobramycin susceptibility, the antibiogram of CI14 and CI17 was unchanged (in both control and UMB-treated cells) while all other strains showed increased susceptibility against TOB30. Moreover, UMB treatment increased the CIP5 susceptibility of CI06, CI17, CI19, CI23, CI24, and PAO1, while the susceptibility of CI14 was decreased (control: 30.67 ± 1.15 mm, UMB treated: 28.33 ± 0.47 mm). The isolates CI06 and CI19 were resistant to the CTX30 and UMB treatment and did not alter the resistant pattern of CI06 and CI19. UMB treatment reduced the K30 sensitivity of CI14 (control: 10 mm, UMB treated: 9.33 ± 0.94) but increased the susceptibility of CI17, CI19, CI23, CI24, and PAO1. The sensitivity pattern of CI06 remained unchanged in both control and treated cells. Further validation is required to determine the susceptibility pattern of strains exhibiting CTX resistance. In this case, MIC of CTX has to be determined before validating the efficacy of combinatorial approach with UMB. Though the sensitivity pattern of test strains varies against selected antibiotics, antibiogram results exhibited the increased susceptibility of each test strain against ≥4 antibiotics in UMB treatment. Hence, the observed results exemplify the efficacy of UMB with antibiotics in combinatorial approach against various test strains.

Furthermore, the observed results of the MTT assay and microscopic visualization in cytotoxicity analysis exemplified the non-deleterious effect of UMB up to 150 µg/ml on HepG2 cell lines. Previous studies have also well-established insights into the pharmacological effect of UMB on animal models. For instance, Ramu et al. (2016) have proved the reduced hyperglycemic condition with the reversal of serum/urine protein abnormalities and urea and creatinine concentration in rat models after *in vivo* administration of UMB at concentrations of 100 mg/kg and 200 mg/kg body weight for 4 weeks. Similarly, the anti-ulcerogenic effect of UMB up to 200 mg/kg with antioxidant property was observed in the Swiss mouse model (Cruz et al., 2020). Taken together, based on all the observed experimental lines of evidence of the present investigation, the present study demonstrates the anti-virulence potential of UMB against the opportunistic pathogen, *P. aeruginosa.*

## Conclusion

The present investigation exemplifies the antibiofilm and anti-virulence efficacy of UMB against *P. aeruginosa.* UMB effectively reduced the formation of characteristic air–liquid interface biofilm of various strains of *P. aeruginosa* on polystyrene and glass surfaces. Functional interaction analysis of differentially regulated proteins confirmed that the majority of proteins coupled to virulence pathways of *P. aeruginosa* are downregulated in treated cells. DAVID server-based GO and gene enrichment analysis and KEGG pathway interpretation revealed that the differentially expressed proteins are related to cellular component, biological process, and molecular function. In particular, the major virulence-associated proteins such as RhlR, LasA, KatA, Tpx, FusA1, Tsf, PhzM, PhzB2, CarB, DctP, MtnA, and MscL were found to be downregulated in UMB-treated cells. In line with this, the phenotypic assay results also revealed the lowered concentration of pyocyanin, protease, elastase, and catalase production in treated cells. Furthermore, the antibiotic susceptibility of the treated cells was found to be significantly enhanced to various classes of antibiotics, exemplifying the combinatorial approach combining antibiofilm and antibiotic agents against *P. aeruginosa* strains. Furthermore, cytotoxicity analysis on HepG2 exemplified the non-toxic nature of UMB to the hepatic cells. Altogether, the study reveals the potential antibiofilm and anti-virulence efficacy of UMB against *P. aeruginosa* strains, and the efficacy has to be further evaluated in higher animal models before considering the UMB for therapeutic applications.

## Data availability statement

The authors acknowledge that the data used in this study are made publicly available. The 16S rRNA gene sequences of clinical isolates were submitted in the GenBank nucleotide database ( https://submit.ncbi.nlm.nih.gov/subs/genbank/) with accession numbers OP740402 (*P. aeruginosa* CI06), OP740403 (*P. aeruginosa* CI14), OP740404 (*P. aeruginosa* CI17), OP740405 (P. aeruginosa CI19), OP740406 (*P. aeruginosa *CI23), OP740407 (*P. aeruginosa* CI24). The LC-MS/MS data used in this study is given in Supplementary Information (Data Sheet1.CSV) and can be downloaded using the provided Supplementary information link. 

## Author contributions

TK and SKP designed the experiments. TK, SB and MN performed the experiments. TK analyzed the data. TK and SKP wrote the manuscript. All authors contributed to the article and approved the submitted version.

## Acknowledgments

The authors thankfully acknowledge the Department of Science and Technology-FIST [Grant No. SR/FST/LSI-639/2015(C)] and UGC-SAP [Grant No. F.5-1/2018/DRS-II(SAP-II)] for providing instrumentation facilities. Financial support to TK provided by the Department of Science and Technology (DST), New Delhi, in the form of the DST-INSPIRE Fellowship (No.: DST/INSPIRE Fellowship/IF170511) is thankfully acknowledged.

## Conflict of interest

The authors declare that the research was conducted in the absence of any commercial or financial relationships that could be construed as a potential conflict of interest.

## Publisher’s note

All claims expressed in this article are solely those of the authors and do not necessarily represent those of their affiliated organizations, or those of the publisher, the editors and the reviewers. Any product that may be evaluated in this article, or claim that may be made by its manufacturer, is not guaranteed or endorsed by the publisher.
