# Supplementary material for: Proteomic profiling spotlights the molecular targets and the impact of the natural antivirulent umbelliferone on stress response, virulence factors, and the quorum sensing network of Pseudomonas aeruginosa
Source: Front Cell Infect Microbiol. 2022 Nov 30;12:998540. doi: 10.3389/fcimb.2022.998540 (PMC9748083; doi:10.3389/fcimb.2022.998540)
Supplement: Supplementary file 2 [file DataSheet_2.zip › Supplementary Information.pdf]

Supplementary Table 1. Details on the primers used to analyse the list of differentially regulated proteins of *P. aeruginosa* PAO1 upon UMB treatment

S. No.	Gene name	Sequence detail 5'→3'
1.	*algL*	algL_F: CCCTCGCCTACCACAACTAT<br>algL_R: CTTGAGGTCGGTCATGTCCT
2.	*clpB*	clpB_F: TTCATCGACGAACTGCACAC<br>clpB_R: CCTTCTCGATGTACTGGCGA
3.	*clpP2*	clpP2_F: ATATCGAGATCTACCGCCGC<br>clpP2_R: ATCTTGTTGACCAGGCCGTA
4.	*dctP*	dctP_F: ACTTCACCGAGTCCAACCAT<br>dctP_R: GATGATCTTCTGCTTGGCCG
5.	*fliD*	fliD_F: GCGAAGACAAGAATCCGGTC<br>fliD_R: TCTTCTCGTCGATCTCCAGC
6.	*fusA1*	fusA1_F: GTTCGGTCATTGCTGGATCC<br>fusA1_R: TGGAGTCGACGTCATGGTAG
7.	*katA*	katA_F: GCTTCTGGGTCAAGTTCCAC<br>katA_R: CTGCACGTACATCTTCCAGC
8.	*lasA*	lasA_F: GCTGAATGACGACCTGTTCC<br>lasA_R: GTCAGCAACACTTTCGGGTT
9.	*mscL*	mscL_F: GGGCAATGTCGTGGATATGG<br>mscL_R: CGGTCTGAATGAACTTGCCA
10.	*phzM*	phzM_F: CGAATTGACCAAGGCCATCC<br>phzM_R: AGCAGGTAGATATCGCCGTT
11.	*rhlR*	rhlR_F: CTCCTCGGAAATGGTGGTCT<br>rhlR_R: TTCTGGGTCAGCAACTCGAT
12.	*sodB*	sodB_F: TGCCTTACGAAAAGAACGCC<br>sodB_R: GGAGGAGCTCTTGACGATCT
13.	*tpx*	Tpx_F: CGTGACCCTGGAAAACTTCG<br>Tpx_R: ACTACGTTCTCCAGGCCTTC
14.	*rpsS*	rpsS_F: AGAGTTTGATCCTGGCTCA<br>rpsS_R: GCACGTGCGAGCATGAAGACG

(1a) **(1b)** **(1c)**

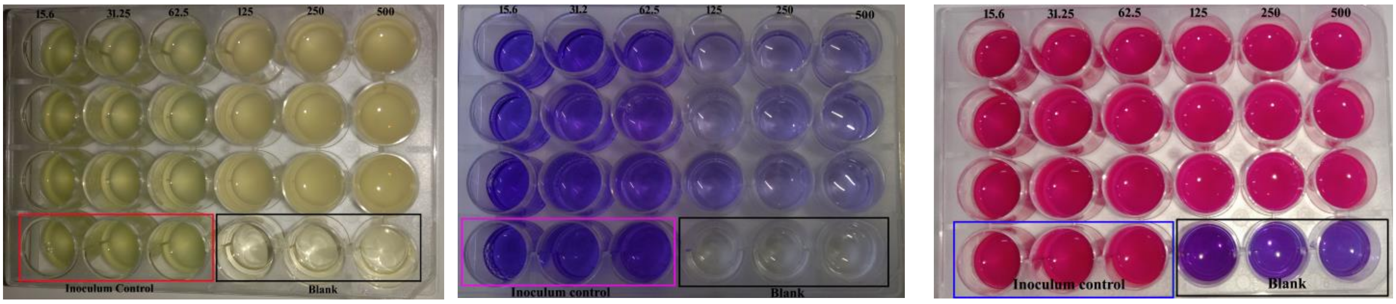

Supplementary Figure 1. 24 well plate assays representing the impact of UMB on growth and biofilm formation of *P. aeruginosa* PAO1. Growth of *P. aeruginosa* after 24 h incubation with 125 µg/mL of UMB (1a). Biofilm absorbed dye eluted with 30% Glacial acetic acid in crystal violet staining assay (1b) Resazurin (blue) is converted to resorufin (pink) in metabolic viability assay [Assay was performed from 24 h incubated culture of *P. aeruginosa* with various concentration of UMB].

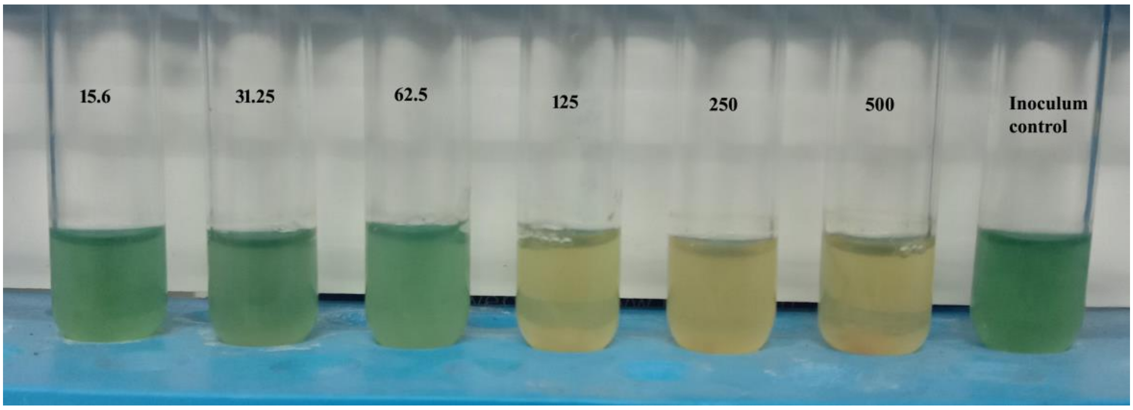

Supplementary Figure 2a. Representative photograph of 24 h grown culture of *P. aeruginosa* PAO1 with various concentration of UMB for ring biofilm assay

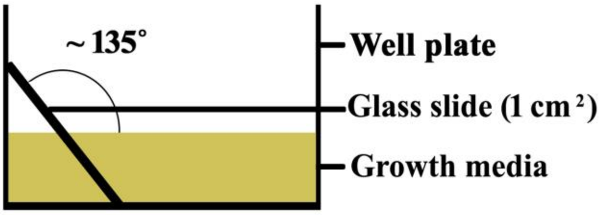

Supplementary Figure 2b. A pictorial representation of 24 well plate assay with glass slides for microscopic analysis of *P. aeruginosa* biofilm. As *P. aeruginosa* has a tendency to form characteristic ring biofilm at air-liquid interface, biofilm easily adheres on glass surface when the slides are positioned slanting to the wells.

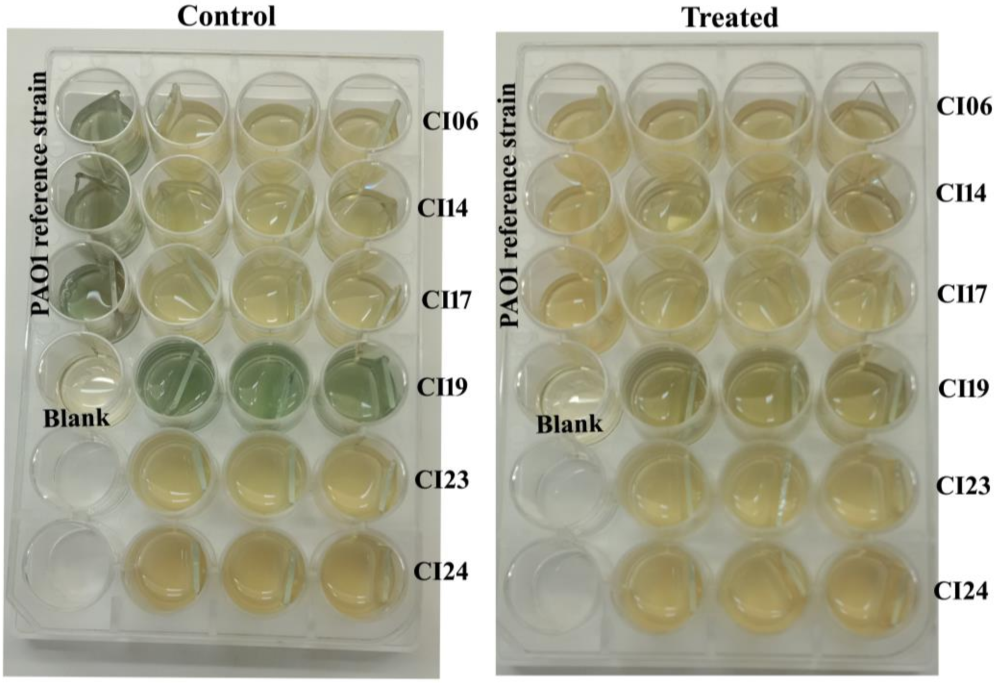

Supplementary Figure 2c. Photograph representing the 24 h grown culture of various *P. aeruginosa* strains. The glass slides were washed with sterile saline and further proceeded with CV staining for microscopic analysis.

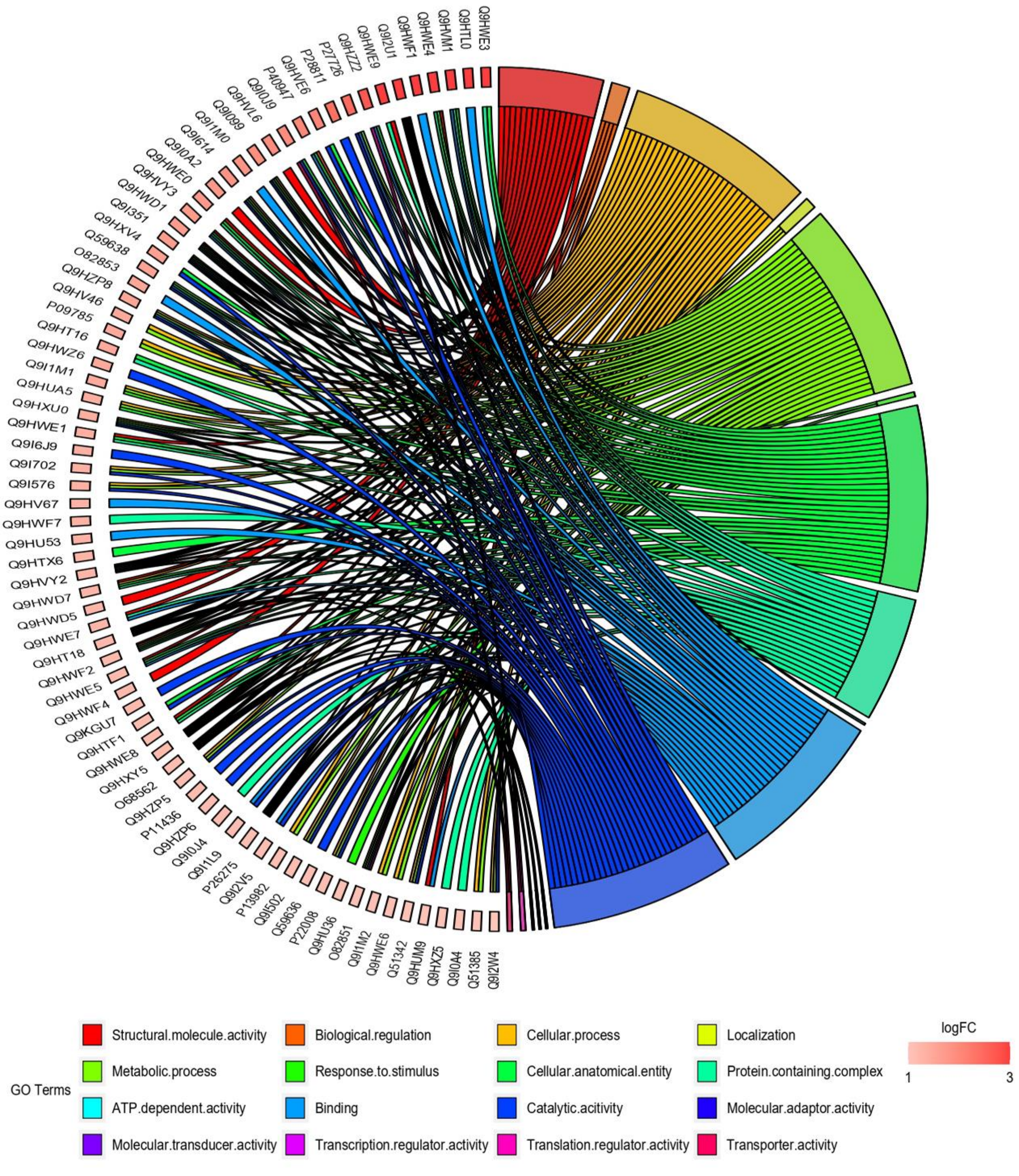

Supplementary Figure 3a. Chord plot representing the functional clusters of upregulated proteins of *P. aeruginosa* PAO1 upon UMB treatment. Enrichment data was obtained from DAVID based gene enrichment analysis.

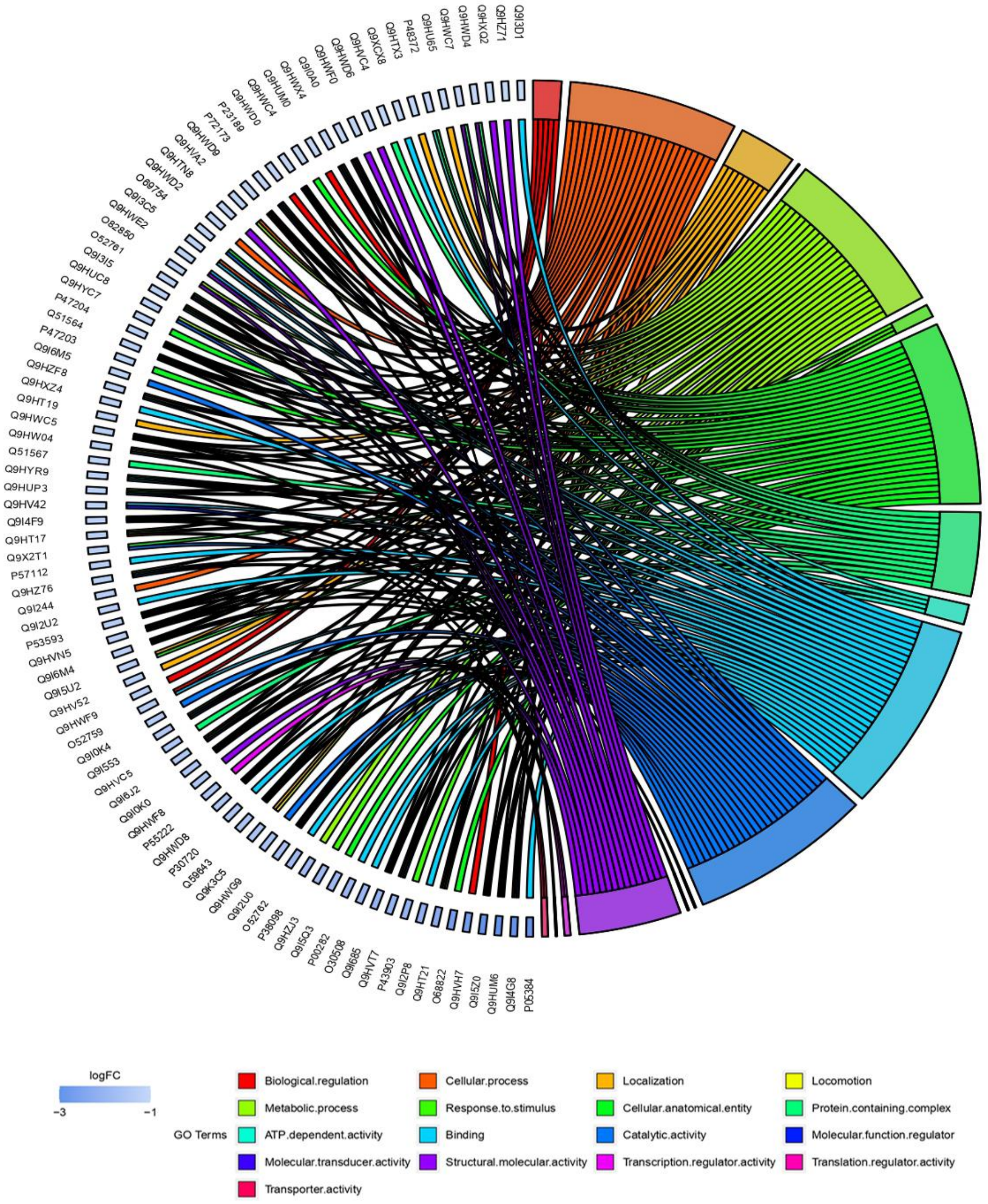

Supplementary Figure 3b. Chord plot representing the functional clusters of downregulated proteins of *P. aeruginosa* PAO1 upon UMB treatment. Enrichment data was obtained from DAVID based gene enrichment analysis.

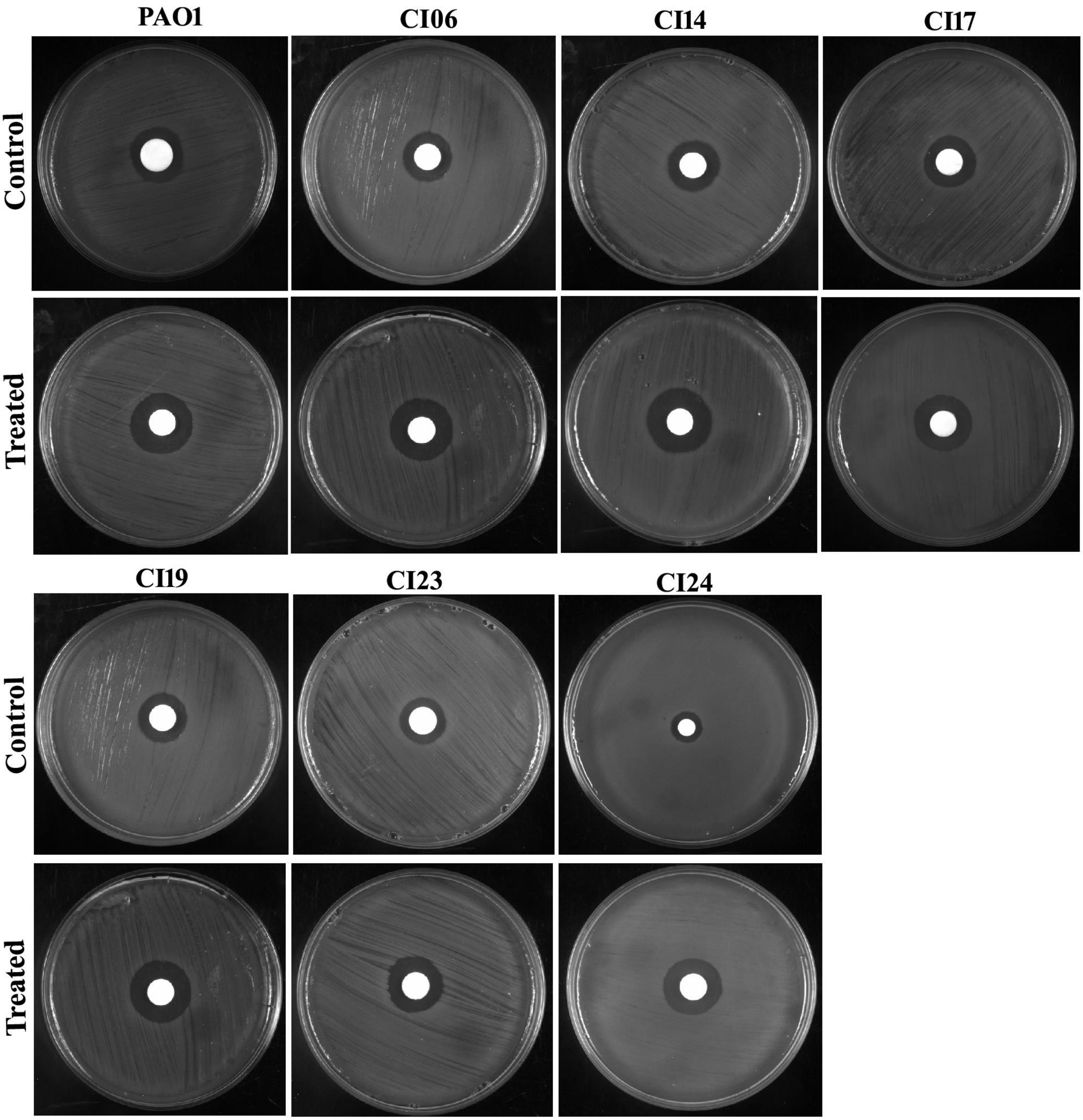

Supplementary Figure 4. Representative image of H_2O_2 sensitivity assay of various *P. aeruginosa* strains. Increased zone of clearance in treated cells than control cells represent the sensitivity of *P. aeruginosa* to the H_2O_2 due to decreased catalase production upon UMB treatment.

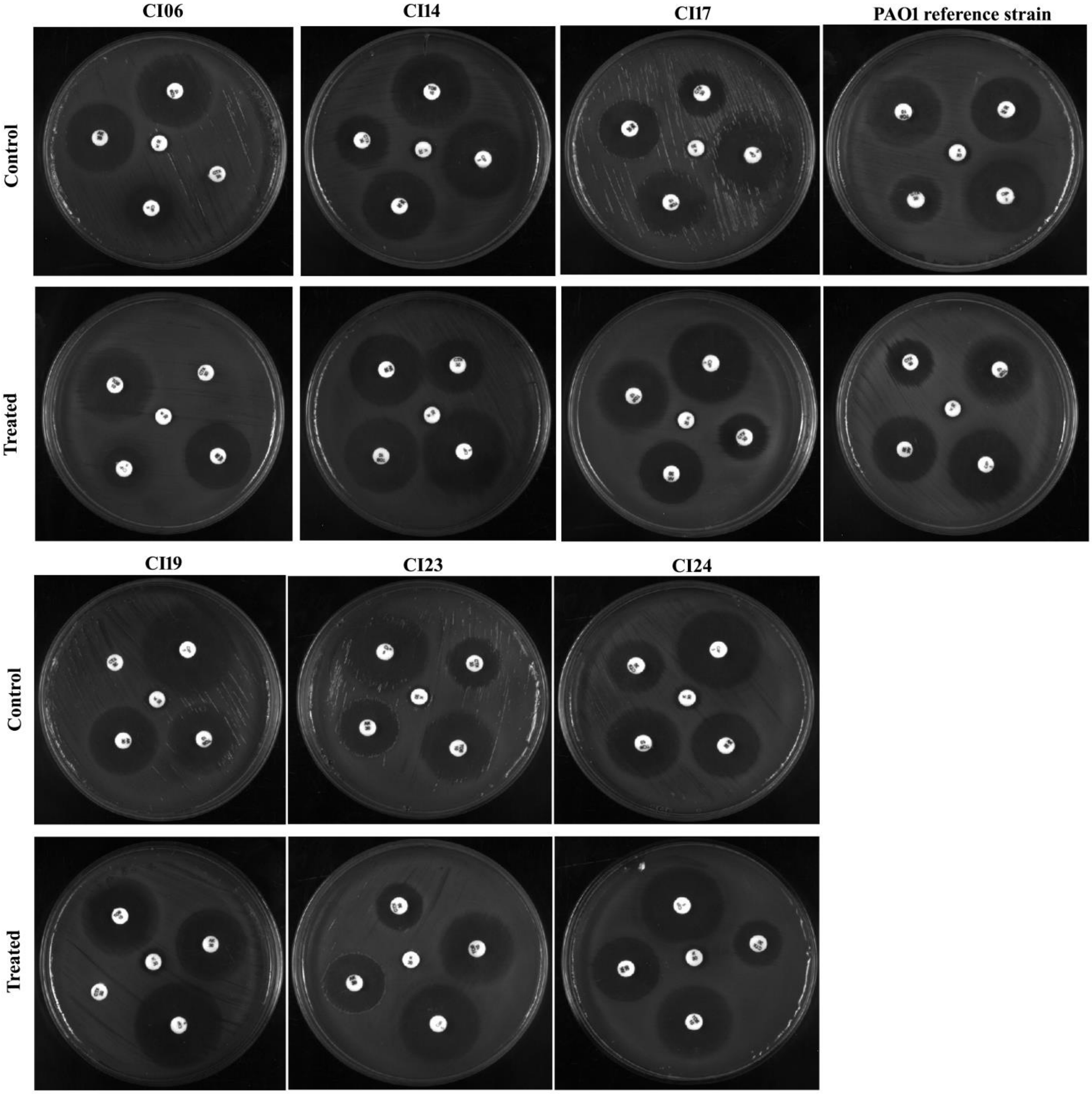

Supplementary Figure 5. Clinical strains showing various antibiotic susceptibility pattern in antibiogram analysis of control and treated groups. The zone of clearance is markedly increased in treated groups.
